# A LEAP Forward in Wildlife Conservation: A Standardized Framework to Determine Mortality Causes in Large GPS‐Tagged Birds

**DOI:** 10.1002/ece3.70975

**Published:** 2025-03-27

**Authors:** Connor T. Panter, Carina Nebel, Maximilian Raab, Verena Strauss, Clara Freytag, Manuel Wojta, Hannah Böing, Patrick Hacker, Rainhard Raab, Jendrik Windt, Annika Posautz, Anna Kuebber‐Heiss, Patrick Scherler, Martin U. Grüebler, Urs G. Kormann, Martin Kolbe, Alexandre Millon, Javier de la Puente, Javier Viñuela, Duncan Orr‐Ewing, Oliver Krone, Torsten Langgemach, Susanne Åkesson, Brady Mattsson, Petra Sumasgutner, Manuel Alcántara de la Fuente, Ernesto Alvarez, Juan Arizaga, Albert Bach Pagès, Ana Bermejo, Guido Ceccolini, Nayden Chakarov, Peter Derpmann‐Hagenström, Marek Dostál, Gerd Fabian, Wolfgang Fiedler, Manuel Galán, Clément Ganier, Andreas Gärtner, Liza Glesener, Alfonso Godino, Zuzana Guziová, László Haraszthy, Caka Karlsson, Katharina Klein, Ivan Literák, Nicolas Lorenzini, Manuela Löwold, Christopher Lüning, Boris Maderič, Karel Makoň, Kerstin Mammen, Ubbo Mammen, Torsten Marczak, Hynek Matušík, Aymeric Mionnet, Sara Morollón, Jakub Mráz, Winfried Nachtigall, Bernd Nicolai, Marta Olalde Fernández, Meinolf Ottensmann, María Jesús Palacios González, Jean‐Yves Paquet, Vladimír Pečeňák, Lubomír Peške, Thomas Pfeiffer, Robert Pudwill, Dušan Rak, Tim Maximilian Rapp, Alexander Resetaritz, Stef van Rijn, Romain Riols, Arturo Rodríguez, Luisa Scholze, Laura Schulte, Aurélie de Seynes, Jan Škrábal, Péter Spakovszky, Eike Steinborn, Ján Svetlík, Samuel Talhoet, Miklós Vaczi, Anne‐Gaelle Verdier, Zdenĕk Vermouzek, Diego Villanúa Inglada, Jörg Westphal, Rainer Raab

**Affiliations:** ^1^ School of Geography University Park Campus, University of Nottingham Nottingham UK; ^2^ School of Applied Sciences Moulsecoomb Campus, University of Brighton Brighton UK; ^3^ Department of Biology University of Turku Turku Finland; ^4^ Turku Collegium for Science, Medicine and Technology University of Turku Turku Finland; ^5^ TB Raab GmbH Deutsch‐Wagram Austria; ^6^ Institute of Materials Chemistry TU Wien Vienna Austria; ^7^ Research Institute of Wildlife Ecology Department for Interdisciplinary Life Sciences Vienna Austria; ^8^ Institute of Wildlife Biology and Game Management, Department of Ecosystem Management, Climate and Biodiversity University of Natural Resources and Life Sciences Vienna Austria; ^9^ Swiss Ornithological Institute (SOI) Sempach Switzerland; ^10^ Rotmilanzentrum Am Heineanum Halberstadt Germany; ^11^ Aix Marseille Univ, CNRS, IRD Avignon Univ, Institut Méditerranéen de Biodiversité et d’Écologie Aix‑en‑Provence France; ^12^ Independent Researcher Madrid Spain; ^13^ Instituto de Investigación en Recursos Cinegéticos (IREC‐CSIC‐UCLM‐JCCM) Ciudad Real Spain; ^14^ RSPB Scotland Edinburgh Scotland UK; ^15^ Leibniz Institute for zoo and Wildlife Research (IZW) Department Wildlife Diseases Berlin Germany; ^16^ Brandenburg State Agency for Environment, Bird Conservation Centre Buckow Germany; ^17^ Department of Biology Lund University Lund Sweden; ^18^ Konrad Lorenz Research Center, Core Facility for Behavior and Cognition University of Vienna Grünau im Almtal Austria; ^19^ Gobierno de Aragon Edificio San Pedro Nolasco Zaragoza Spain; ^20^ GREFA Madrid Spain; ^21^ Department of Ornithology Aranzadi Sciences Society San Sebastián Spain; ^22^ Ecological and Forestry Applications Research Centre (CREAF) Bellaterra Catalonia Spain; ^23^ Departament de Biologia Animal, Biologia Vegetal i Ecologia Universitat Autònoma de Barcelona Bellaterra Catalonia Spain; ^24^ CERM Endangered Raptors Centre Association Rocchette di Fazio Italy; ^25^ Department of Animal Behaviour Bielefeld University Bielefeld Germany; ^26^ Joint Institute for Individualisation in a Changing Environment (JICE) University of Münster and Bielefeld University Bielefeld Germany; ^27^ LIFE EUROKITE External Assistant Gifhorn Germany; ^28^ Department of Biology and Wildlife Diseases, Faculty of Veterinary Hygiene and Ecology University of Veterinary Sciences Brno Brno Czech Republic; ^29^ LIFE EUROKITE External Assistant Uelzen Germany; ^30^ Max Planck Institute of Animal Behavior Radolfzell am Bodensee Germany; ^31^ LPO France, Fonderies Royales Rochefort CS France; ^32^ Naturschutzsyndikat Sicona Luxembourg Luxembourg; ^33^ Acción Por el Mundo Salvaje (AMUS) Villafranca Spain; ^34^ Ochrana Dravcov Na Slovensku Raptor Protection of Slovakia (RPS) Bratislava Slovakia; ^35^ Pro Vértes Természetvédelmi Közalapítvány Hungary; ^36^ Natur&ëmwelt ASBL Kockelscheuer Luxembourg; ^37^ LPO AURA‐Loire Saint‐Étienne France; ^38^ Mitteleuropäische Gesellschaft Zur Erhaltung der Greifvögel Deutschland (MEGEG DE) Fulda Germany; ^39^ LEA LandesEnergieAgentur Hessen GmbH Wiesbaden Germany; ^40^ Des op Záchranná Stanice živočichů Plzeň Plzeň Czech Republic; ^41^ ÖKOTOP–Büro für Angewandte Landschaftsökologie Halle (Saale) Germany; ^42^ Förderverein für Ökologie Und Monitoring von Greifvogel– und Eulenarten e.V. Halle (Saale) Germany; ^43^ LIFE EUROKITE External Assistant Bützow Germany; ^44^ LIFE EUROKITE External Assistant Březolupy Czech Republic; ^45^ LPO Champagne‐Ardenne Ferme Des Grands Parts Outines France; ^46^ SEO/BirdLife Madrid Spain; ^47^ LIFE EUROKITE External Assistant Planá nad LužnicíLužnicí Czech Republic; ^48^ Förderverein Sächsische Vogelschutzwarte Neschwitz eV Neschwitz Germany; ^49^ Arabako Foru Aldundia–Diputación Foral de Álava Vitoria‐Gasteiz ES Spain; ^50^ Biological Station Gütersloh/Bielefeld Bielefeld Germany; ^51^ Directora de Programas de Conservación Dirección General de Sostenibilidad de la Junta de Extremadura Mérida Spain; ^52^ Département Études Natagora Namur Belgium; ^53^ LIFE EUROKITE External Assistant Praha Czech Republic; ^54^ LIFE EUROKITE External Assistant Weimar Germany; ^55^ LIFE EUROKITE External Assistant Germany; ^56^ ANITRA System s.r.o. Praha Czech Republic; ^57^ Deltamilieu Projecten Vlissingen the Netherlands; ^58^ LPO AURA Auvergne Clermont‐Ferrand France; ^59^ Asociación Hontza Salvatierra‐Agurain (Araba) Spain; ^60^ Department of Behavioural Ecology Bielefeld University Bielefeld Germany; ^61^ LPO France Direction Territoriale Aquitaine Avenue de la Gare Saint‐Jean‐pied‐de‐port France; ^62^ LPO Occitanie Palmas‐d’Aveyron Aveyron France; ^63^ Fertő‐Hanság Nemzeti Park Igazgatóság Sarród Hungary; ^64^ Czech Society for Ornithology Praha Czech Republic; ^65^ Navarra Environmental Management (GAN‐NIK) Pamplona Spain; ^66^ District of Lippe Lower Nature Conservation Authority Detmold Germany

**Keywords:** bird crime, cause of death, GPS tracking, human‐wildlife conflict, population monitoring, survival analysis, wildlife conservation

## Abstract

Anthropogenic activities threaten many wildlife populations by increasing mortality rates, making it crucial to identify the locations and causes of mortality to inform conservation actions. Technological advancements, such as GPS satellite tracking, enable precise recording of wildlife movements. High‐resolution data from such devices can facilitate rapid carcass recovery and provide insights into the mortality causes of tagged individuals. Obtaining required information to determine these causes is complex, and standardized approaches can overcome these limitations. In this study, we introduce the LIFE EUROKITE Assessment Protocol (LEAP), a framework for determining the timing, locations, and causes of mortality in GPS‐tagged birds. LEAP is a multifaceted approach that integrates: (1) GPS tracking data, (2) evidence from the mortality location (site investigation), and (3) necropsy results to derive the mortality cause and a corresponding certainty score. We supplement the detailed description of LEAP with case studies assessing its effectiveness. Using 329 deceased GPS‐tagged red kites ( *Milvus milvus* ) we compared conditions of the carcasses processed using LEAP with 145 opportunistically collected raptor carcasses. We also show that LEAP improves carcass condition and therefore allows for higher quality necropsy results. Additionally, we assessed how availability among sources of information (tracking, site investigation and necropsy) influences the quality of mortality assessments. Applying LEAP with all data sources provided the highest quality assessments in 64% of cases. Some 35% of cases were of high quality without necropsy, instead drawing evidence only from tracking data and site investigations. Predation related mortality was less prevalent (11%) when relying on necropsy compared to cases without necropsy (36%), while poisoning showed the opposite trend. Furthermore, we provide guidelines and empirical examples of mortality assessments. Our standardized LEAP approach ensures the best use of all available information regarding mortality events in GPS‐tagged birds and advances wildlife mortality research as a valuable tool for conservationists and wildlife managers.

## Graphical abstract

A novel framework for determining the causes of mortality of large GPS‐tagged birds, the LIFE EUROKITE Assessment Protocol (LEAP), was developed. LEAP integrates GPS tracking data, site investigation, and necropsy to derive mortality causes and a corresponding certainty score. Two case studies demonstrated improvements in mortality assessments compared to opportunistically collected carcasses.

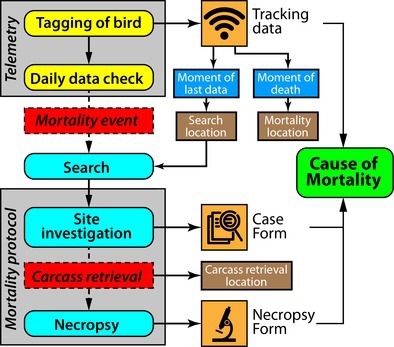

## 1 Introduction

Understanding the impacts of human activities on wildlife is essential for improving our knowledge of anthropogenic threats to wild animal populations (Ceballos, Ehrlich, and Dirzo 2017; Finn et al. 2023; Munns 2006; Prakash and Verma 2022) and is fundamental for successful conservation interventions (Brooks et al. 2006; Prakash and Verma 2022). Data relating to causes of mortality in wild vertebrates can facilitate enhanced understanding of the negative consequences of human–wildlife interactions (Hill, DeVault, and Belant 2019) and trigger appropriate mitigation measures (Ceballos, Ehrlich, and Dirzo 2017; LaDue et al. 2021).

The only widely accepted method to identify the cause of mortality in wild animals beyond reasonable doubt is necropsy. This, however, is associated with multiple problems: Whether a cause of death can be identified strongly depends on the freshness of carcasses and contextual information about the location of death and what was discovered there (Valverde et al. 2020). As deceased animals are often only found by chance and sometimes long after death (Bellan et al. 2013; Langgemach et al. 2023; Stevens and Dennis 2013), obtaining reliable results from necropsy is unlikely in many mortality cases. Even if fresh carcasses are discovered, opportunistic finds can introduce biases toward specific mortality causes. For example, overrepresentation of anthropogenic mortality causes due to higher detectability over natural causes (Mullineaux 2014; Mullineaux and Pawson 2023; Panter et al. 2022). Systematic misidentification of the mortality cause can occur when the chance to quickly retrieve a carcass viable for a necropsy is low. A typical example of this is when a predator moves the carcass to inaccessible areas (DeVault et al. 2011). Similarly, after an animal is illegally killed, it is in the interest of the offender to immediately hide or destroy the carcass, which may result in underestimation of this mortality cause (Murgatroyd et al. 2019). Investigating and addressing these biases is important to reliably distinguish mortality causes, but this is challenging when relying on necropsies alone. Another disadvantage of applying only necropsy is that when an animal dies through trauma, it is difficult to determine what caused the trauma. These factors pose challenges for gaining accurate knowledge of mortality causes in wildlife, especially when working with species that traverse large distances or occur at low population densities and therefore have low recovery rates (McClure et al. 2018).

Near real‐time tracking of wild individuals using Global Positioning Systems‐Global Systems for Mobile Communications (GPS‐GSM hereafter “GPS”) technology has been increasingly used to study wildlife movements and behavior (e.g., Baert et al. 2018; Bergen et al. 2022; Kays et al. 2021). This technology can provide data on the location of mortality events and facilitate the retrieval of fresh carcasses of monitored animals after the point of death (Klaassen et al. 2014; Pérez‐García et al. 2023; Sergio et al. 2018; Serratosa et al. 2024). One of the main advantages of this tracking technology is that the probability of detecting a given mortality cause may be higher than that of other methods. This may be more effective for certain mortality causes than for others, that is, it may be easier to detect cases of disease or predation than events where human‐facilitated concealment has occurred. Despite this, using GPS tracking data can improve precision and accuracy in mortality assessments. In addition, utilizing available information from tracking data such as movements before and after death, combined with investigations of mortality sites, may enable more reliable assessments of mortality causes than solely relying on necropsy results. For example, the characteristics of the surrounding environment, (e.g., the presence of anthropogenic structures) can inform the origin of trauma during necropsy.

Through GPS tracking information, it might be possible to ascertain illegal activities including shooting (Brochet et al. 2016; Katzner et al. 2020; Thomason, Wallen, and Katzner 2023) and poisoned baits (Christensen, Lassen, and Elmeros 2012; Green, Pain, and Krone 2022; Mateo‐Tomás et al. 2012; Molenaar et al. 2017; Ogada 2014). These are still prominent despite legal protection, and often go unpunished due to a lack of evidence (Thomason, Wallen, and Katzner 2023). Here, GPS tracking information can provide evidence that facilitates successful detection and prosecution of the perpetrator. While field‐based mortality assessments are often unavailable or inconclusive by themselves, necropsies conducted on fresh carcasses can be used to determine causes of mortality more confidently by relying on the skills and experience of trained veterinary pathologists (Cooper 2021). Although the combination of these information sources can be used to increase the accuracy of mortality causes, there is neither a widely accepted framework of how to integrate different information sources into the identification of mortality causes in birds, nor a reliable way to determine mortality causes in cases where no conclusive necropsy can be performed.

An approach to determine mortality causes in ungulates based on a combination of satellite tracking data, inspection of the carcass site, and necropsy has been recently published (Cristescu et al. 2022). However, there is currently no comprehensive guidance on determining mortality causes in birds that account for predator communities and anthropogenic threats that are unique to flying animals, e.g., wind turbine collisions or electrocution. Existing approaches to ascertain mortality causes in raptors focus on toxicology. These include national guidelines (e.g., SAGIR in France https://www.ofb.gouv.fr/) along with international protocols (e.g., Espín et al. 2021) which focus on how to handle carcasses to identify poisonous substances or contaminants. An integrated and standardized approach, which goes beyond the identification of contaminants (e.g., including predation, shooting and collisions) and allows the user to determine mortality locations and ascribe a level of certainty associated with each decision, would improve in‐situ conservation of large birds.

**FIGURE 1:**
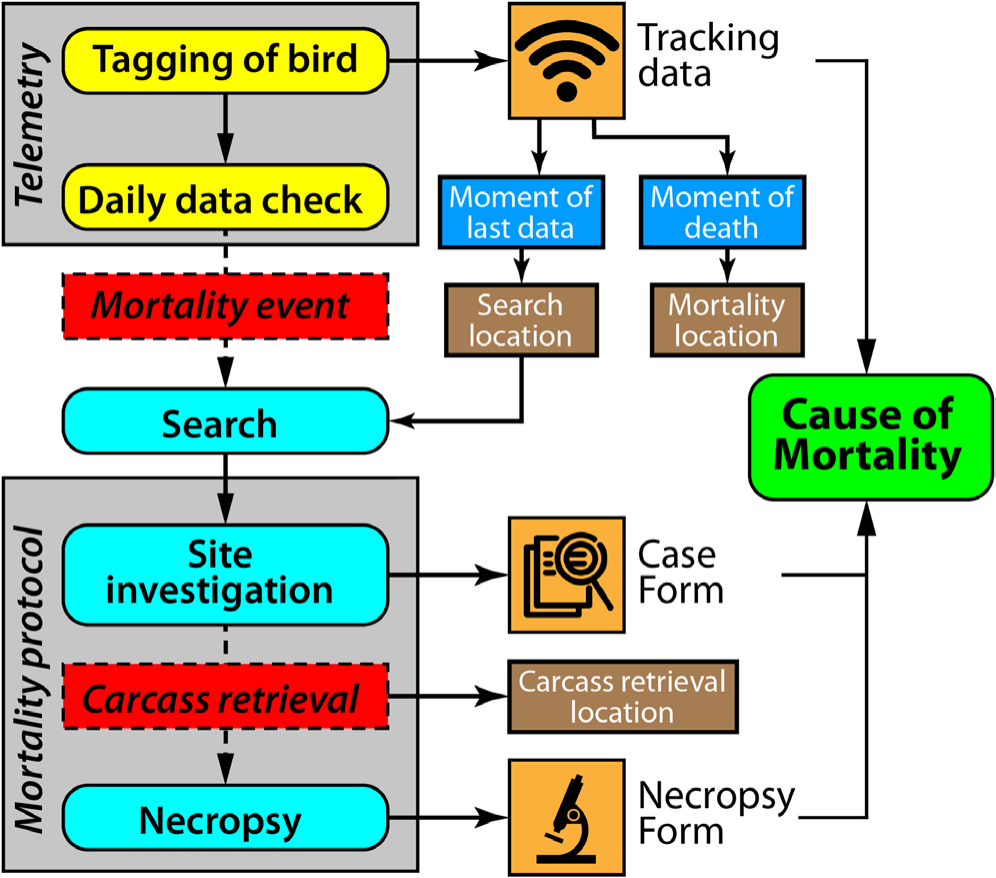
Schematic representation of the process to determine the cause of mortality of GPS‐tagged red kites (*Milvus milvus*) as part of the LIFE EUROKITE assessment protocol (LEAP): Data collection starts with tagging the bird, followed by daily data checks for signs of mortality. If a mortality event occurs, tracking data provides an accurate moment and location of death. This triggers searching for a fresh carcass quickly, with the search location determined from the last known tracking data point. A fresh carcass provides a better chance of carrying out a successful necropsy evaluation. The mortality cause is determined based on three sources: (1) tracking data, (2) observations during the site investigation (documented in the Case Form) and (3) necropsy results (documented in the Necropsy Form). The coloring reflects the following categories: Yellow—GPS tracking; red—event related to the focal carcass; cyan—performed actions during the investigation; orange—information sources for the assessment; blue—point in time; brown—location; green—result of assessment.

Here, we describe and assess the LIFE EUROKITE Assessment Protocol (LEAP), which was developed using the red kite ( *Milvus milvus* ) as a model system with the following goals: (1) Fast identification of mortality events to facilitate rapid carcass retrievals for high‐quality necropsy analyses; (2) obtain a representative and minimally biased overview of the prevalence of various mortality causes by identifying those that might otherwise go undetected or remain inconclusive; and (3) providing clear and detailed guidelines for the integration of multiple sources of information besides necropsy, allowing better determination of mortality causes in GPS‐tracked birds. To achieve these goals while maximizing the level of certainty in determining the cause of mortality, the LEAP framework integrates all available sources of data (Figure 1): (i) GPS tracking locations and their metadata depicting kite movements before, during, and after death, (ii) environmental and biological evidence present at the location of death, and (iii) findings from necropsies. LEAP accounts for the availability and quality of data by incorporating an estimation of the certainty surrounding the mortality cause decision. This certainty assessment provides an important layer of transparency when making inferences from patterns in mortality data (Molinari‐Jobin et al. 2012).

To facilitate the application of LEAP in a standardized way, we provide accompanying documentation to perform GPS tracking surveillance, site investigations, and necropsies, and present guidelines on how to interpret the obtained data to identify the most likely cause of mortality, along with assigning a level of certainty. We investigate how LEAP performs relative to other approaches and show that it improves carcass condition at retrieval time and increases the number of conclusive mortality assessments. We show that even when necropsy is unavailable, assessments from tracking data and site investigations may provide reliable mortality cause estimates in many cases. We highlight potential biases in previous studies by showing that predation is detected significantly less often when only considering necropsy results. Using these comparisons, we propose that LEAP is a suitable unified framework with sufficient potential to improve research on avian mortality and to inform conservation actions in a wide range of species.

## 2 Methods

We first introduce the LEAP framework (section 2.1), before providing an overview of the LIFE EUROKITE project, which developed and applied LEAP using GPS‐tagged red kites across Europe (section 2.2). Then we describe the sampling design of our case studies and statistical approaches (section 2.3). Key terms used throughout are defined in the glossary Appendix A. In the Appendix, we illustrate the assessment process when determining mortality causes using LEAP on the basis of eight exemplary cases.

### 2.1 The LEAP Framework

In the following subsections, we initally provide a detailed overview of the three data sources that feed into LEAP: GPS tracking (2.1.1), site investigations, and subsequent carcass retrieval and handling (2.1.2). Next, we describe how carcass retrieval allows a necropsy to be carried out by a professional and trained veterinary pathologist (2.1.3). Finally, we introduce certainty classes that maximize transparency when assigning mortality causes (2.1.4).

#### 2.1.1 LEAP Part 1: GPS Tracking

The LEAP process starts with the tagging of birds, which makes this method independent of opportunistic findings and provides a comparatively representative and accurate assessment of mortality causes. Tracking data from modern GPS transmitters is a valuable information source that can provide insights when determining the moment of death, mortality location, carcass location, and causes of mortality. Modern GPS transmitters provide accelerometer and pitch/roll data, allowing for assessments of the bird's behavior before, during, and the movement of the carcass after the moment of death. As the transmitter typically continues to function even after the death of the tagged bird, it also provides spatio‐temporally explicit data about scavengers moving the carcass or feeding from it, or cases when humans intentionally move the carcass, as often observed in cases involving bird crime (Box 1). Exceptions are when the tag is destroyed by the event causing the death, such as a collision, or is moved underground or to any site with poor signal reception by a predator, scavenger or human. All tags should contain contact information (email and phone number) and requests to return the GPS tags in case they are found.

**FIGURE 2:**
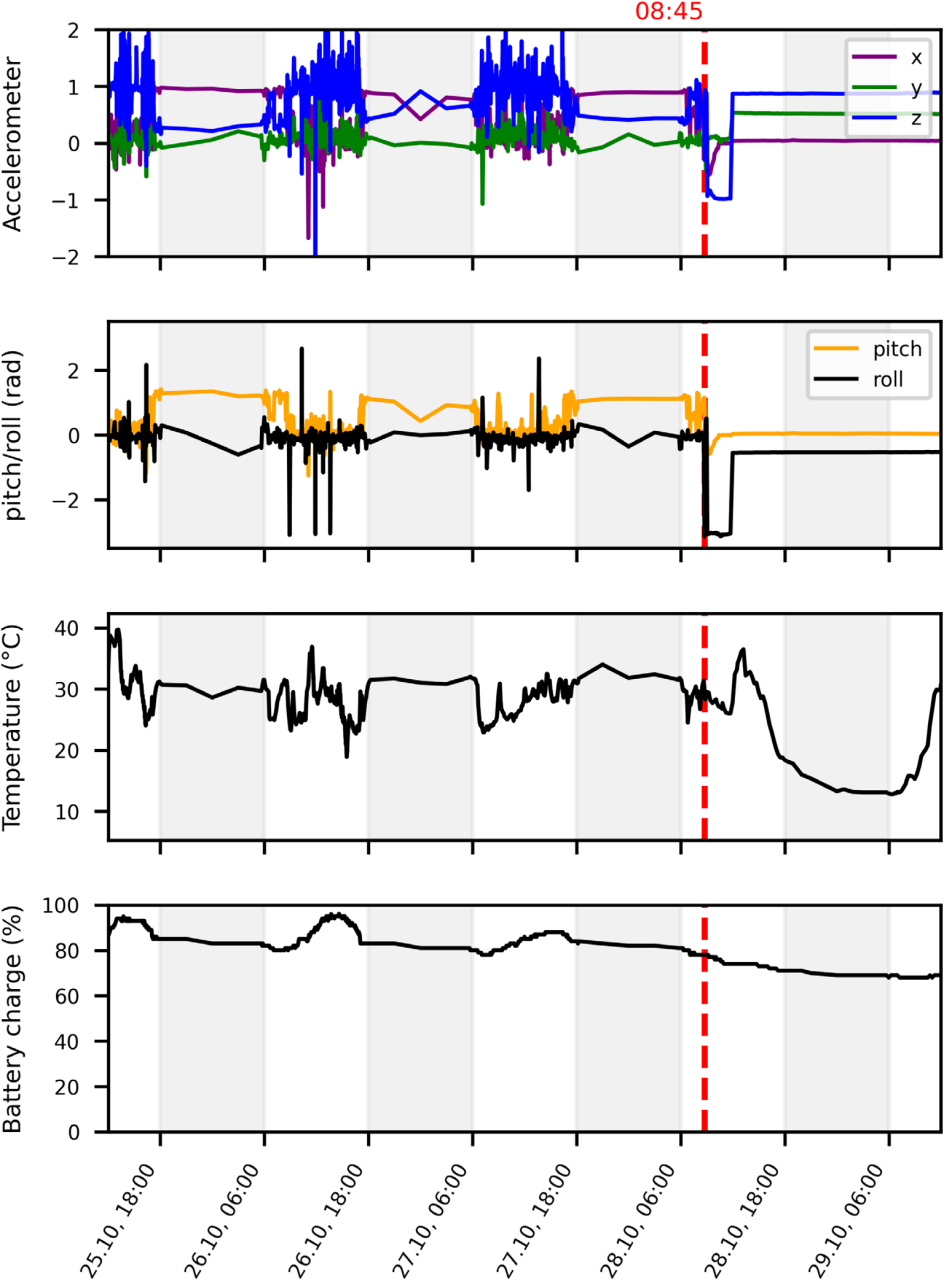
Tracking metadata from red kite (*Milvus milvus*) RK_2159 (road collision victim): The four plots show the recorded tracking metadata between October 25 at 12:00 and October 29 at 12:00, transmitted once every 5 h. Gray shaded areas represent nighttime, white shaded areas daytime. The moment of mortality is marked with a red, dashed vertical line on the morning of October 28 at 8:45.

The surveillance of all tagged birds is carried out by the tracking data coordinator(s) at intervals of every second day to up to three times a day using online web platforms provided by satellite transmitter companies. Frequent checks of tag position and metadata (Figure 2) are necessary to allow fast detection of mortality events. Healthy birds will show a distinct pattern reflecting their daily activity rhythms in the GPS transmitter metadata.

Depending on weather and season, the temporal resolution of the GPS‐GSM locations typically ranged between one data point per hour to one data point per second, subject to device settings and remaining battery charge. High resolution might be required to identify some types of mortality (e.g. differentiate predation from scavenging, see Box 2). We recommend at least one data point per 5 minutes. Resolution of the GPS data may temporarily decrease for several reasons including low‐light conditions (particularly at higher latitudes and during certain parts of the day), and the effects of duty cycles which may not align with a birds' death and the tag's active period. In these situations, the frequency of data retrieval will decrease, for example, to one data point every few hours or even gaps lasting days or weeks, with knock‐on impacts on carcass freshness required for necropsy.

During the day (white areas in Figure 2), fluctuations in accelerometer data indicate movements and typical three‐dimensional transmitter orientations. At night (shaded areas), movement activity is reduced and the transmitter orientation remains the same as the bird is perched. The occurrence of a mortality event often renders the bird into an unnatural position, causing sharp spikes in accelerometer and pitch/roll data. After death, cessation of all movement activities of the bird is indicated by clear differences to when the bird was alive in the parameters collected by the GPS transmitter, such as flat lines (Figure 2). While the bird is alive, temperature measurements mainly show a mixture between external and body temperature, which is in most cases closer to body temperature. As soon as the bird is dead, the temperature reflects ambient temperature based on season and weather and oscillates following a daily cycle. The battery charge decreases if the tag's solar panel is not adequately exposed to sunlight.

When a mortality event is detected by the tracking data coordinator(s), the responsible local partner is immediately alerted and informed about details concerning the death. They then send out site investigators to commence the ground search. To make the search most effective, the searcher is provided with the last known locations of the bird and the location of mortality, as well as the Mortality Protocol, a guide on how to perform the search and documentation (see File S1 and box in Figure 1). Some transmitter manufacturers (e.g., Anitra) also offer tools to optimize the search process, providing a centroid point of the last recorded locations to increase accuracy. Other alternative tracking technologies can also be used, including the ARGOS system, which relies on a Goniometer and signal strength to locate actively transmitting tags (see Cioffi et al. 2023). However, the accuracy of ARGOS transmitters is generally lower than that of GPS trackers.

In the case of suspected poisoning incidences, the GPS data can be used to backtrack the movements of the bird before it died to determine the location of the potential poison bait. This allows confirmation of poisoning incidences and facilitates evidence collection. The transmitter metadata may also allow differentiation between predation and scavenging or identification of illegal activities that contributed to the bird's death (see below). Case examples where we demonstrate how modern GPS tracking data can be used in mortality assessments can be found in Appendix B.

#### 2.1.2 LEAP Part 2: Site Investigations

Site investigations often provide valuable information about the cause of mortality. As circumstances can be difficult to interpret, it is crucial to document evidence and collect data on the conditions in the field with accompanying high‐quality photographs. This also allows a reassessment of mortality cases at a later point in time when additional information becomes available.

Many subtle signs can facilitate an identification of the mortality cause, and the following examples are not exhaustive: In collision cases, blood might be visible, for example, on wind turbine rotor blades after a collision or on the ground after the collision with a car. Collisions with windows or glass surfaces may also include body or wing impressions. The site investigation must consider potential illegal activities (such as illegal shooting) or cases of poisoning (details listed below, see Box 1). In many situations, a likely mortality cause is readily identifiable in‐situ (e.g., collision with a vehicle), and the suspected cause of death along with detailed evidence must also be entered by the site investigator in the Case Form (see File S2).

Besides visiting the mortality location in person, information can also be gained from an online assessment through tools such as Google Maps and Google Earth, especially via street view mode. However, caution needs to be applied as online images can be outdated and should never substitute a proper in‐person site investigation.

We recommend that users bring the following material into the field: printed Case Form copies, a map, a mobile phone with GPS and camera including the contact number(s) of local police or potential wildlife crime units, labels, and pens. If the carcass can be collected, a printed permit, sturdy plastic zip‐locked bags of different sizes (one should be large enough to fit a whole carcass, smaller ones may be used to collect samples of feathers or body parts), solid containers with tightly sealable lids (to prevent any leaks or contamination), cooling packs (one or two), a paper box (for transportation via courier services), and plastic gloves. If the carcass must be transported across jurisdictions within a country, vet clearance may be required. For many species, a Convention on International Trade in Endangered Species of Wild Fauna and Flora (CITES) permit is required when transferring carcasses across international borders.

The Case Form helps to record all necessary information (see File S2), including a written description of the following: mortality location, characteristics of the surrounding environment with supporting photographic evidence, collected samples, and further steps initiated (e.g., informed authorities). Photographs of the site surrounding the carcass should include a panorama showing any nearby infrastructure and close‐up photographs of the following: the carcass or remains, GPS tag, signs of struggle, and any evidence relating to illegal activity along with any seized objects (e.g., gun cartridges or poisoned baits). In the Case Form, a presumed cause of death is noted if such assessment is possible in the field.

When the carcass or GPS transmitter is located, accurate GPS coordinates are recorded. A site investigation is carried out to document the mortality as thoroughly as possible at both the retrieval location and the mortality location (whenever they are not the same and even if no carcass/transmitter is found), as well as the suspected mechanism (e.g., scavenging or moved by humans) of any inferred movement from death to retrieval location. Site investigations are also performed at locations where poison baits are suspected. In the case of such suspected movement, additional evidence for this is recorded. The ground area is searched within > 100 m of the carcass or GPS transmitter for any abnormalities that could indicate bird crime (refer to the points laid out in Box 1).

For a successful necropsy, the carcass must be delivered to a laboratory as soon as possible. Considering legal issues, the collection of a carcass, especially for species of conservation concern, might be illegal or require special permission, e.g., from the landowner or government authority. This can lead to difficulties as it might not always be possible to retrieve the carcass for necropsy, depending on national laws. We recommend contacting government authorities to learn about relevant laws and necessary permits. Establishing contacts with relevant authorities (i.e., in all areas where mortality might occur) at the beginning of a study ensures efficient carcass retrieval.

Site investigators must always wear protective plastic gloves when touching carcasses or any evidence such as baits or poisonous substances. There might be specific requirements such as restricted handling of carcasses where highly pathogenic avian influenza (HPAI) or other contagious diseases are present (Simancas‐Racines et al. 2023). Carcasses and other evidence are collected in sealable bags, and a second bag for HPAI suspect carcasses is used. During transport, the carcass is kept at a cool temperature to preserve the biological material, for example, with two cool packs.

If the carcass is delivered to a necropsy laboratory within 48 h, freezing should be avoided until necropsy examination has taken place. Freezing can limit the efficacy of any pathological analyses that may be performed on the carcass, for example, microbiological analyses, gross pathology, or histology of organs and tissues (Valverde et al. 2020). Tags should be removed before long‐term freezing, as this may damage the internal functioning of the devices. If the carcass cannot be delivered to a pathology laboratory within 48 h, it must be frozen as soon as possible to prevent further autolysis. Necropsy can then still be conducted at a later point, albeit with a reduced reliability (Valverde et al. 2020).

In cases where poisoned baits are found, they are placed in solid containers and sealed tightly to prevent leakage or contamination. All samples and evidence are labeled with permanent marker pens. Evidence and collection bags contain a label with at least the following information: (1) date, (2) species/evidence, (3) number of samples, (4) locality, (5) name and contact information of the sender/collector, and (6) case code from the Case Form. Any collection document or record that is in use by local authorities in the country is also completed separately from the documents in this protocol.

Box 1 **Bird crime: is the case suspicious?**

Suspected poisoning (including direct and indirect poisoning) and illegal shooting (both considered as “bird crime”) are often challenging to identify. If illegal activities are suspected to play a part in the bird's demise, alert the local police and wait for their arrival before moving the carcass or any evidence such as traps or other dead birds. In cases where suspected illegal activities have resulted in the death of a bird, the police should collect samples, starting a well‐grounded chain of custody of evidence that will be useful in a court trial. Do not touch the GPS transmitter or carcass in such cases.

Evidence of illegal activities may be present on the carcass itself or the GPS transmitter, or characteristics of the wider environment may indicate that illegal activities have taken place. Important points to consider when performing a site investigation include:

1. On the GPS transmitter, focus on the straps that mount the transmitter on the back of the bird. Have the straps been cut off (knife, scissors), bitten off, or loosened naturally? If the strap has been cut off, this is a strong indication that the bird was killed by illegal activities, although it has to be kept in mind that some bite marks can also appear as very clean cuts.
2. The GPS transmitter might be smashed or damaged.
3. If a bird has been poisoned, there may be little to no specific abnormalities present in the field. Inspect the beak and nose of the bird; blood, leftover feed (e.g., prey remains) or unnatural colorations on the beak, can indicate poisoning. Carbofuran is typically indicated by its blue, lilac or purple color. Aldicarb presents as hard black granules. Poisoned birds might have cramped legs and claws, traces of vomit present, and a twisted neck (Molenaar et al. 2017). Several raptor or corvid carcasses may be close to each other, indicative of mass poisoning. In the surrounding area, you might find a lack of necrophagous insects, dead animals (including necrophagous insects, dogs or foxes) or suspicious items in the field, e.g., poison bait material like meat or eggs.
4. If the bird has been trapped illegally, e.g., glue traps or snares, check whether the traps are still present within the immediate area.
5. Footprints, vehicle tire marks, or trampled vegetation in the surrounding area may be present if the bird died through illegal activities.
6. If nestlings were illegally taken from a nest, the tree where the nest is located may have damaged bark or broken branches.

Using data supplied by the GPS transmitters can allow the identification of illegal activities, such as shooting, from the way the carcass is moved by the perpetrator shortly after its death: movement tracks following roads after the mortality signal was detected, indicating the tag has been transported in a vehicle, potentially being at constant temperatures (indicating transportation inside a vehicle or storage inside a building or cooler box). Such observations in the tracking data are also communicated to the site investigator when being informed of the mortality case by the tracking data coordinator(s). In Appendix B4, we present two cases in which kites were illegally shot and their carcasses could not be retrieved, likely because their bodies were disposed of, and a third case where a kite was killed by the banned poison carbofuran (Commission of the European Communities 2007/416/EC).

In addition, cases of Stop No Malfunctions (SNM) can occur when a bird has been illegally killed (often shot), with the tag and bird disposed of to hide the crime (Ewing et al. 2023; Murgatroyd et al. 2019; Whitfield and Fielding 2017). These cases are characterized by a sudden end in transmission, in absence of any earlier signs of tag malfunction (e.g., decline in battery performance). SNMs occurred within our sample of tagged red kites (*n* = 28, additional to the number presented in the case study examples), but it is often not clear how many can be attributed to illegal activities. We analyzed each suspected SNM on a case‐by‐case basis and decided to exclude these from our data set due to the lack of sufficient information, and to avoid overrepresenting illegal killing.

#### 2.1.3 LEAP Part 3: Necropsies

Necropsy, which is performed by a professional pathologist or qualified veterinarian (Brownlie and Munro 2016), is a reliable source of information to identify the physiological contributors to the cause of mortality (Kagan 2016), which can then be set into context with information gained from site investigations and GPS tracking. Necropsies are important in determining mortality causes, although they are not required in all cases. Necropsies may also allow insights into secondary causes of death (e.g., diseases or malnutrition, which can result in the bird becoming the victim of a predation event) that would otherwise remain unknown.

Necropsies are sometimes performed with preconceived assumptions surrounding potential causes of death. On the one hand, this can lead to a biased assessment of mortality. On the other hand, contextual information can be essential, for example, in cases of poisoning to test for the suspected substances. Otherwise, necropsy can accurately and independently conclude that a death was caused, for example, by blunt force trauma. This assessment can be further refined by site investigations, which allow an identification of a road collision as the most likely cause of the trauma. Inspecting a carcass allows assessments of secondary mortality causes such as disease or poisoning. We provide the LEAP Necropsy Form (see Supporting Information), facilitating the standardization of procedures and laboratory tests across different professionals, laboratories, and studies. In case the police are involved, they may initiate formal investigations and will often provide only the final diagnosis to the researchers.

Being able to perform a conclusive necropsy requires locating carcasses quickly, which is supported by GPS tracking data alongside daily surveillance of GPS data within the LEAP framework. If necropsies are not possible due to advanced decomposition of the carcass, removal of the carcass by a scavenger or human, or the carcass being located within inaccessible terrain, GPS transmitter metadata becomes invaluable alongside photographic evidence of the carcass and its immediate environment.

#### 2.1.4 Identifying Cause of Mortality and Level of Certainty

Mortality causes and associated levels of certainty are assessed by a group or single individual with expertise in the ecology of the target species, using available evidence from the three information sources: GPS tracking, site investigation, and necropsy. Here we briefly describe the following levels: conclusive—split into certain, probable, possible—and inconclusive. We use the term “conclusive” to include cases varying in confidence about the mortality cause from those that we are certain of (i.e., high confidence) to those that are possible (i.e., moderate confidence) based on the evidence provided. The assessor identifies the most likely cause of mortality from the main categories (e.g., predation, poisoning) from the master list (given for kites as an example in Appendix Table C1) and associated level of certainty, along with providing a reason for each assignment based on the data types used. There is also the option to select more detailed subcategories, for example, predation by an eagle, poisoning by carbofuran, in case such additional information is available. Certainty levels are always chosen based on the aforementioned main categories. Here, we provide guidelines on determining the level of certainty. To illustrate the certainty assessment, we present eight cases in Appendix B and explain in detail how mortality causes and certainty levels were determined. Additionally, we provide detailed examples of evidence leading to each certainty level in Appendix C.

#### “Certain” Mortality Cause

A case is evaluated as “certain” if there is unequivocal proof of the mortality cause. A typical example is when the carcass condition allows for an unequivocally conclusive necropsy with a clear result pointing to a single cause of mortality, which is also in accordance with tracking and site investigation data.

Although the aim should always be to use information from all three LEAP sources for cause of death assessments, the carcass might be unretrievable or necropsy can fail, for example, due to the carcass being frozen before the necropsy. If a necropsy is unavailable or inconclusive, a case can also be considered certain based on observations during site investigations and tracking data alone. Assuming that the carcass is found, it can provide a clear indication of mortality (e.g., signs of struggle and a plucking site would indicate predation, blood on a rotor blade and amputated wing would indicate collision with the wind turbine); then the certain category may be applied even with no necropsy. If a carcass cannot be retrieved, a case can also be considered certain based on observations during site investigations and tracking data alone (sudden death in an open area followed by movement along streets would indicate shooting and subsequent movement of the tag and/or carcass inside a vehicle).

#### “Probable” Mortality Cause

The tracking data or the site investigation strongly point to a single cause of death, but the definitive proof is missing. There is, however, no evidence for any other cause of death. Poisoning or disease can lead to atypical behavior of the kite in the hours and days prior to its death, for example, the bird moves less and sits idle for consecutive days before mortality occurs.

#### “Possible” Mortality Cause

There are indications for a likely cause of death, but other plausible mortality causes cannot be completely ruled out due to incomplete information, for example, where the carcass is in poor condition or unavailable (thus necropsy analysis is impossible), and only the site investigation and GPS tracking provide indications of a specific mortality cause. Often, possible mortality causes remain plausible due to limited information gained from transmitter metadata at the moment of death. An example of such limitations is when a mortality event happens during a period with gaps in the GPS tracking data.

#### “Inconclusive” Mortality Causes

Cases where the mortality cause cannot be determined with sufficient confidence, as none of the above categories apply, should be classified as inconclusive. This can occur when no carcass or signs of the bird are found in site investigations, the frequency of tracking data is too low to show what occurs at the time of death, or there are multiple top‐ranked causes of which none is more likely than the others based on available information.

Box 2 **LEAP for distinguishing predation from scavenging following differing causes of death**

The inability to distinguish between occurrences of predation and scavenged individuals following other types of mortality (e.g., collision with infrastructure) remains a challenge for mortality assessments (Cristescu et al. 2022). This results in a two‐fold conundrum when determining mortality causes from a carcass: (1) predation might be overlooked due to the difficulty of interpreting the carcass condition; and (2) scavengers may have moved the carcass away from the actual location of death, which may compromise or eliminate contextual evidence (e.g., proximity to poisoned bait or infrastructure) to inform the mortality assessment. When applying LEAP, the GPS location data and the associated metadata can help differentiate predation from scavenging. Examples of such evidence include strong pitch/roll fluctuations indicating plucking or feeding (i.e., predation of the tagged bird) and movement of the carcass immediately after death indicating scavenging. Retracing the mortality location after scavengers moved the carcass allows one to investigate the mortality site and find evidence of the mortality cause. In Appendix B3, we present two predation events and summarize the conclusive evidence with guidelines on how to differentiate predation from scavenging.

### 2.2 Overview of the LIFE EUROKITE Project and Tagging of Red Kites

Data for the case studies presented here originated from the LIFE EUROKITE project (LIFE18 NAT/AT/000048, https://www.life‐eurokite.eu), a large network of non‐governmental organizations, scientists, veterinarians, and volunteers operating across most of continental Europe. During this project, 1,251 red kites were tagged up until 2022 in 14 European countries with solar‐powered GPS‐GSM satellite tracking transmitters. Devices were sourced from Anitra (https://anitracking.com/), Ornitela (https://www.ornitela.com/), Ecotone (https://ecotone‐tracking.com/index.php/en), e‐obs GmbH (https://e‐obs.de/) and Interrex (https://interrex‐tracking.com/). Transmitter weights varied between 20 and 28 g (2%–3% of the birds' average weight of *ca.* 962.1 g) and were fitted as harnesses on the back of the kites using Teflon ribbon spanning 9–11 mm in width (Kenward 1985). Comprehensive use of LEAP began in 2019, so we used all birds that died between 2019 and the end of 2022. We observed 423 mortality events, of which 329 birds died after leaving the nest. Here we describe two empirical case studies using these data along with information from necropsies and site investigations to examine the performance of LEAP.

### 2.3 Case Studies Data Collection and Statistics

For the first case study, we used the 329 kite mortality cases of birds that were tagged within the LIFE EUROKITE project and died after fledging in 18 countries (Table C2). The cases were processed following LEAP with one or more of the three focal information sources: tracking (T), site investigation (S) and necropsy (N). Of these individuals, 104 also underwent necropsy examination (TSN) with the remaining 225 being assessed using only GPS data and site investigation information (TS). We compared the 104 TSN carcasses with 145 carcasses (from 16 raptor species, Table C3) that were collected opportunistically and delivered for necropsy (non‐LEAP‐N) to the Research Institute of Wildlife Ecology, University of Veterinary Medicine Vienna (Austria) between 2012 and 2018. First, we compared the condition of carcasses retrieved with the help of tracking information to those retrieved opportunistically. To do this, we modeled the probabilities of each carcass condition using both methods, that is, LEAP vs. non‐LEAP‐N. In R version 4.3.2 (R Core Team 2023), we ran a multinomial log‐linear model from the package “nnet” (Venables and Ripley 2002) and fitted “carcass condition” (i.e., “very good”, “good”, “medium”, “bad”, or “very bad”, detailed descriptions and examples are presented in Table C4) as the response variable and “process” (i.e., TSN vs. non‐LEAP‐N) as the explanatory variable. A Type II ANOVA was run to test for group‐level significance of the explanatory variable. Unless otherwise noted, only statistically significant comparisons (*p* < 0.05) are reported for both case studies.

In addition to assessing whether carcass condition improved following LEAP, we also explored how the level of certainty when assessing a mortality cause differed using TSN or TS vs. non‐LEAP‐N. To do this, we used a Chi‐squared Test for Independence and compared the distribution of “conclusive” and “inconclusive” certainty levels assigned to each case for non‐LEAP‐N, with those systematically monitored and gathered using LEAP (TSN and TS). For the LEAP cases, we used the LEAP certainty classes “certain”, “probable”, “possible”, (subclasses of “conclusive” [of various confidence]) and “inconclusive” (e.g., due to the carcass being unretrievable or in very bad state, 2.1.4). As opportunistically collected carcasses underwent necropsy but were not subjected to the full LEAP process, we could not distinguish among all three levels of uncertainty. Instead, mortality causes were assessed as “conclusive” or “inconclusive”. For the LEAP cases, we used the LEAP certainty classes “certain”, “probable”, “possible”, and “inconclusive.”

For the second case study, we assessed the same sample as in the first case study, but only considering cases for which a minimally conclusive assessment was obtained (certainty is “certain”, “probable”, or possible”, LEAP‐pooled, *n* = 299). Using LEAP, we explored whether the mortality cause assessments were dependent on whether necropsy examinations were available. We compared cases for which a necropsy was performed (TSN, *n* = 97) with those without necropsy (TS, *n* = 202).

We specifically focused on poisoning (*n* = 69), predation (*n* = 83) and shooting/trapping (*n* = 31) cases because there may be a bias toward the carcass not being retrieved due to removal by scavengers, predators, or wildlife‐crime offenders. We compared whether the availability of necropsy (i.e., TSN vs. TS) explained differences in the probabilities of three causes of mortality: poisoning, predation, and shooting/trapping. For all other mortality causes, we categorized them as “other” (*n* = 116). We fitted a multinomial log‐linear model by using cause of mortality as the response variable and availability of necropsy (i.e., TSN vs. TS) as a binary explanatory variable. A Type II ANOVA was run to test for the significance of the explanatory variable. We also compared the frequency distribution among mortality causes for TSN carcasses to that for all red kites tagged in the LIFE project (LEAP‐pooled).

## 3 Results and Discussion

In describing our findings from the first case study (section 3.1), we summarize how carcass condition and classification of certainty levels regarding mortality causes improve by following LEAP. In the second case study (section 3.2), we show how evaluating carcasses by only using necropsy or adding the LEAP framework changes the proportion of specific determined mortality causes (predation, poisoning and illegal shooting/trapping).

### 3.1 Case Study 1: Level of Certainty Based on Evidence of Mortality Cause

**FIGURE 3:**
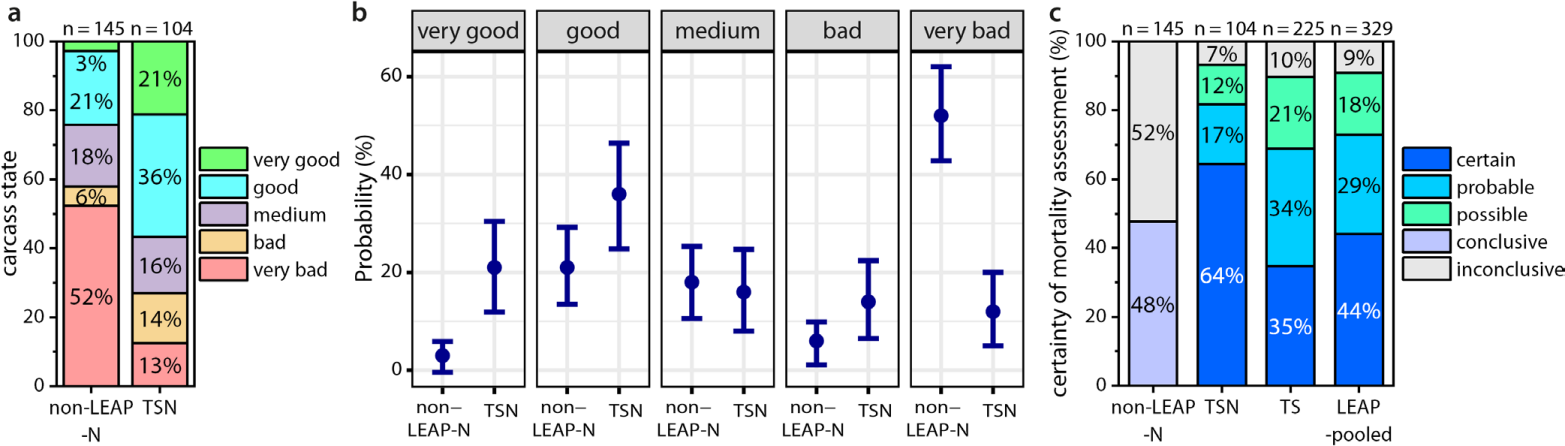
Comparison of 145 opportunistically collected raptor carcasses (non‐LEAP‐N) and 329 post‐fledging red kite (*Milvus milvus*) carcasses collected and assessed using TSN. (a) Comparison of the carcass conditions (classified as “very good”, “good”, “medium”, “bad”, and “very bad”). (b) Modeled effects, with 95% confidence intervals, of process on carcass condition for 104 kite carcasses of TSN and 145 carcasses of non‐LEAP‐N. (c) Comparison of the certainty assigned to each mortality assessment based on information sources tracking (T), site investigations (S) and necropsy (N). The certainty classes are conclusive and inconclusive for non‐LEAP‐N, for kites analyzed within the LEAP framework, conclusive is split by certain, probable, and possible.

We show that carcass condition significantly improves if carcasses were collected following LEAP (TSN) than when they were opportunistically collected and only assessed via necropsy (Figure 3a,b). This is primarily evident by the higher “very good” and “good” carcass conditions (TSN: 21% and 36%, respectively; non‐LEAP‐N: 3% and 21%, respectively) and the high proportion of very bad carcass conditions (Table 1).

**TABLE 1:** Tukey *post hoc* contrasts comparing the condition of carcasses between opportunistically collected raptors and red kite (*Milvus milvus*) carcasses collected and assessed using LEAP.

Carcass condition	Estimate	SE	df	*t*	*p*
**Very good**	**0.184**	**0.042**	**8**	**4.35**	**0.002**
**Good**	**0.142**	**0.058**	**—**	**2.45**	**0.040**
Medium	−0.016	0.048	—	−0.33	0.751
Bad	0.089	0.039	—	2.26	0.053
**Very bad**	**−0.399**	**0.053**	**—**	**−7.58**	**< 0.001**

*Note:* Opportunistically collected raptors of 16 species (*n* = 145, Table C2) were collected in Austria from 2012–2018 and underwent necropsy (non‐LEAP‐N). Red kite carcasses (*n* = 104) were collected across 18 countries (Table C2) from 2019–2022 that underwent necropsy (N) but also had information on tracking and site investigation (TSN). SE = standard error, df = degrees of freedom. Significant contrasts highlighted in bold.

The improvement of the carcass condition compared to opportunistically collected carcasses is primarily due to the tagging of the birds, which allowed the application of LEAP. Only through LEAP and the daily surveillance of tagged birds for mortality events, the carcass condition can be improved due to faster recovery times.

Certainty levels ascribed to the mortality causes increased in carcasses with information from tracking, site investigation, and necropsy (TSN) relative to those collected opportunistically with only necropsy (non‐LEAP‐N), with almost two‐thirds of cases (64%) ruled as certain and only 7% as inconclusive (Figure 3c and Table 2). The proportion of conclusive assessments was higher using TSN compared to using non‐LEAP‐N (*X* ^*2*^ = 61,504; df = 1).

**TABLE 2:** Comparisons of levels of certainty assigned to each mortality cause, percentage in brackets.

Procedure	Number of carcasses (%)					Total
	Certain	Probable	Possible	Conclusive	Inconclusive	
Opportunistically collected carcasses						
Non‐LEAP‐N	—	—	—	70 (48)	75 (52)	145
LIFE EUROKITE assessment protocol						
TSN	67 (64)	18 (17)	12 (12)	97 (93)	7 (7)	104
TS	78 (35)	77 (34)	47 (21)	202 (90)	23 (10)	225
LEAP‐pooled	145 (44)	95 (29)	59 (18)	299 (91)	30 (9)	329

*Note:* Information sources are tracking (T), site investigation (S) and necropsy (N). Comparisons were made using 329 red kite (*Milvus milvus*) carcasses from LEAP collected across 18 countries (Table C3) from 2019–2022, of which necropsies were performed for 104 cases, and 145 carcasses from 16 raptor species (Table C3) opportunistically collected in Austria from 2012–2018 that underwent necropsy (non‐LEAP‐N). “LEAP‐pooled” = all LEAP carcasses (TSN and TS) combined.

The increase in certainty is based on two improvements: First, enhanced carcass condition for better necropsy results; second, the availability of additional information from the tracking data and observations from the site investigation facilitated a more conclusive assessment (Figure 3c and Table 2).

When comparing the full LEAP (TSN) to only using information from tracking and site investigations (TS), we found a reduction in the proportion of “certain” assessments and an increase of “probable” and “possible” cases (Figure 3c and Table 2). A substantial proportion (35%) of TS carcasses were evaluated as “certain” and only 10% remained inconclusive. Despite TSN being better (i.e., having a higher proportion of certain cases) when determining mortality causes than TS, there was still a significant increase in conclusive assessments when using TS compared to non‐LEAP‐N (*X* ^*2*^ = 136,161; df = 1; *p* < 0.0001). This demonstrates that methods that exclude necropsy can also be useful for determining the cause of death in many instances.

In summary, we show that necropsy, which relies on carcasses in fresh and good condition, is an important contributor to high certainty mortality assessments. Using necropsy in synergy with tracking information and well‐documented site investigations can further improve the certainty of the cause of mortality. This allows for more confident analyses of mortality causes in large birds.

### 3.2 Case Study 2: Determined Mortality Cause Based on Availability of Necropsy

**FIGURE 4:**
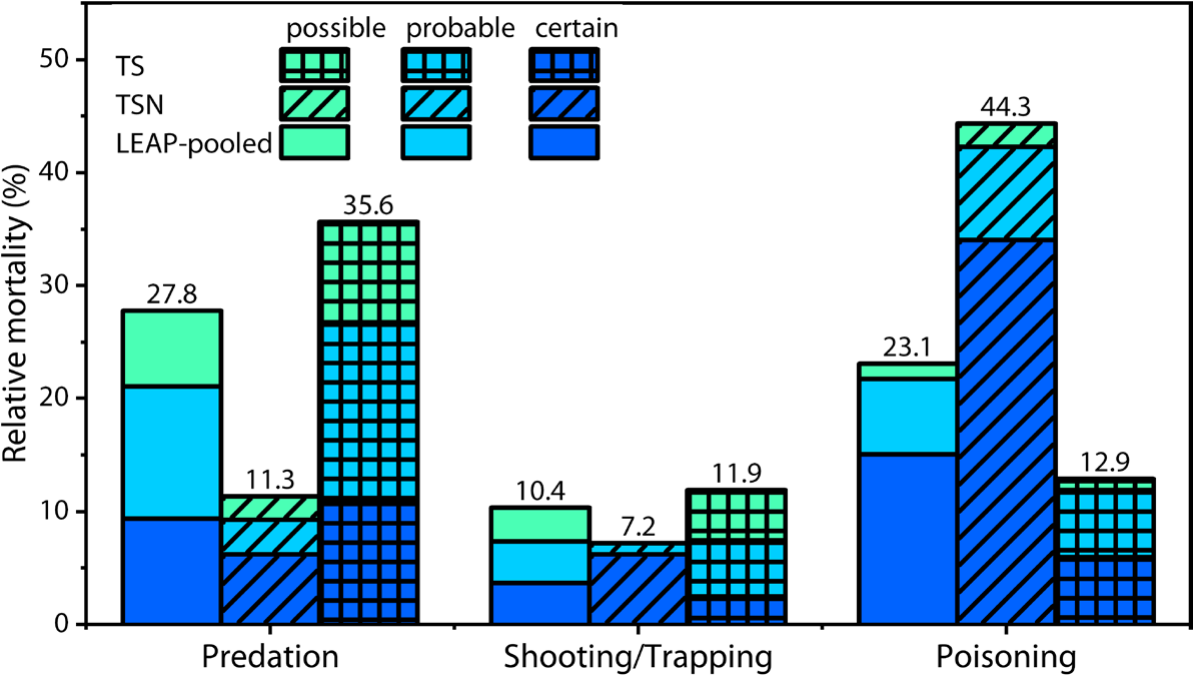
Causes of mortality for red kites (*Milvus milvus*). Left: LEAP‐pooled (*n* = 299, solid colors), including all cases analyzed using LEAP, middle; TSN (with all three sources of data, including necropsy, *n* = 97, hashed), right; TS (without necropsy, *n* = 202, gridded). The colors indicate the certainty of the assessment, the numbers above the bars show the combined percentage of relative mortality.

There was an effect of the availability of necropsy, that is, TS (without necropsy) versus TSN (with necropsy), on modeled probabilities of mortality causes (*X* ^*2*^ = 43.66) (Table 3). Predation cases were less frequent in TSN (11.3%) compared to TS (35.6%). Predation was the assigned cause of mortality in 27.3% of LEAP‐pooled carcasses (Figure 4). These findings support the existence of a bias in detecting predation when only considering cases where only necropsy is available. Likely, predators and/or scavengers reduce the likelihood of carcass retrieval by moving the carcass to difficult‐to‐access areas. Our results also imply that predation‐caused mortality is likely underrepresented in most mortality studies that rely on necropsy of opportunistically collected carcasses (Bellan et al. 2013; Stevens and Dennis 2013; Langgemach et al. 2023; Naef‐Daenzer et al. 2017).

**TABLE 3:** Tukey *post hoc* contrasts comparing the probabilities of poisoning, predation, shooting/trapping and other mortality cases based on information used for the assessment (TS—tracking and site investigation versus TSN with additional necropsy) for 329 red kite (*Milvus milvus*) carcasses collected across 18 countries (Table C2) from 2019–2022.

Mortality cause	Estimate	SE	df	*t*	*p*
Other	0.025	0.060	6	0.42	0.692
**Poisoning**	**−0.315**	**0.056**	**6**	**−5.65**	**0.001**
**Predation**	**0.243**	**0.047**	**6**	**5.21**	**0.002**
Shooting/trapping	0.047	0.035	6	1.34	0.228

*Note:* Significant contrasts highlighted in bold.

Abbreviations: SE, standard error; df, degrees of freedom.

We found cases where shooting was evident based on GPS tracking data, but the carcass could not be retrieved due to the offenders likely destroying or hiding the carcass and/or tag. Although this might mean that the probability of recovering shooting victims might be reduced, there was no significant difference between TSN (7.2%) and TS (11.9%) for shooting/trapping.

Deaths through poisoning were significantly more frequent in TSN (44.3%) than in TS (12.9%). This is explained by necropsies almost always being performed if poisoning is suspected. The proportion of poisoning was higher if all cases were considered (LEAP‐pooled, 23.1%) compared to TS. Collating data on poisoning cases is essential for effective conservation; however, to obtain a representative perspective on mortality causes, it is important to understand how the availability of necropsy impacts inference.

For predation and shooting/trapping, there were more “probable” and “possible” cases within LEAP‐pooled compared to TSN data as some mortality causes in the former additional cases could only be determined based on the GPS locations, especially when no carcasses were retrieved. By contrast, when shooting victim carcasses were retrieved, necropsy data remained very reliable in identifying this mortality cause as gunshot wounds, bullets, and bullet fragments tend to be easily documented.

Based on our findings, LEAP makes multiple improvements compared to other approaches, including the quality of carcasses upon retrieval and a higher level of certainty when assigning the cause of mortality. We also found that GPS tracking, site investigations, or a combination of these allows for determining the cause of mortality when no necropsy can be performed. For cases without necropsy, a higher proportion of predation cases was found compared to those where no necropsy was performed.

## 4 Applications, Limitations and Future Work

LEAP can be applied to raptors and other large birds capable of carrying a solar‐powered GPS transmitter. However, there are some limitations that should be carefully considered when using GPS data to inform mortality assessments: GPS effectiveness might be limited for species in light‐deprived environments, with insufficient sunlight for battery charging (Silva et al. 2017). Some conditions, including areas of poor network connectivity, can hinder data transmission and carcass retrievals. Data resolution and frequency vary with the transmitter make and model. With technological progress, these problems will likely be resolved. As higher temporal and spatial resolution of tracking data becomes available, this will further enhance the applicability and usefulness of the LEAP approach. Consideration should be given to the effects of carrying the tags on the likelihood of mortality. We did not specifically investigate this aspect, but previous research concluded no effects of satellite telemetry tags on the survival of the closely related black kite ( *Milvus migrans* ) (Sergio et al. 2015). Recommended transmitter weights in relation to body mass vary between 2% and 5% (Cochran 1980; Fuller et al. 2005); our transmitters fall within these recommended values, averaging between 2% and 3% of the birds' body weight. Despite this, future research should explore the effects of carrying transmitters fitted as harnesses on the likelihood of mortality in red kites.

LEAP improves the detection of illegal activities such as poisoning and illegal shooting. Poison baits might be found via GPS tracks, whereas shooting victims can be found even if they were moved or hidden by offenders (if not destroyed). Despite the legal protection of large birds in many countries, illegal killing, especially of raptors, persists globally (Brochet et al. 2016; Balmori 2019; Katzner et al. 2020; Thomason, Wallen, and Katzner 2023). Cases that are successfully prosecuted are limited due to a lack of evidence (Nurse 2015). Therefore, identifying cases of illegal killing and collecting evidence can provide the basis for effective enforcement of wildlife protection laws. LEAP provides an essential tool for prosecuting illegal shooting and illegal poisoning.

As successful carcass retrievals depend on functioning GPS transmitters, there is a potential to introduce bias in mortality studies if tags stop working suddenly. In our data, 35 of 1,251 tags suddenly stopped working, without prior indications of malfunctioning. We have two explanations for these apart from technical failure and suspect that the sudden tag stops might be associated with the death of the bird. First, transmitters may be destroyed upon impact with human infrastructure or with vehicles. Second, the transmitter might have been deliberately destroyed following illegal activities like shooting. Inspecting GPS tracking data can sometimes distinguish these types of failures, although thorough testing of this is required. When a tag is destroyed, typically only information collected before the destruction (i.e., when the bird is still alive) is available.

If LEAP is applied within a broad network of collaborators, this introduces potential biases due to variability in collaborators' reliability, expertise, and resources, which can affect data consistency and accuracy. Conducting LEAP repeatedly on the same set of carcasses and by independent assessors with identical training while excluding one or more data sources in each iteration (e.g., GPS tracking) allows for validation of the advantages we identified in combining data for mortality assessments. LEAP provides a standardized protocol to maximize consistency when applied by a network of collaborators. Therefore, communication and synchronization of the collaborators, as well as carefully following the LEAP guidelines throughout the project, are essential.

## 5 Conclusions

We present the LEAP framework and illustrate its advantages over approaches that use a single type of data for determining causes of mortality: (1) fast detection of mortality events and carcass retrievals, resulting in better carcass conditions, with 73% being “very good”, “good” or “medium” compared to 42% for opportunistic findings, allowing substantial improvement in the performance of necropsy. (2) Obtaining representative mortality data unbiased from locations of opportunistic searches for carcasses, while enhancing transparency through certainty criteria. (3) Associated with the better carcass condition and comprehensive information from site investigations, GPS tracking, and necropsy, we further show an improvement in the certainty of mortality assessments (with 93% being conclusive, and 64% being in the best certainty class). Even in the absence of necropsy information, site investigations and GPS tracks allow 90% conclusive assessments of the cause of mortality, with 35% being in the best certainty class. Considering the many potential causes of mortality and associated evidence to consider, such assessments are very challenging. LEAP provides a concise set of guidelines to account for multiple lines of evidence in determining a cause of mortality and the level of uncertainty.

Understanding the causes of wildlife mortality and their prevalence is crucial for informing conservation strategies, identifying emerging threats, and preserving biodiversity (Ceballos, Ehrlich, and Dirzo 2017; Finn et al. 2023; LaDue et al. 2021; Prakash and Verma 2022). By increasing transparency and consistency in methodology, we can more reliably determine and compare the factors that contribute to wildlife deaths across time and space (Munns 2006; Prakash and Verma 2022). Such improvements in mortality studies can mitigate human impacts on wildlife and inform targeted interventions to protect threatened species. Additional knowledge of wildlife mortality helps assess ecosystem health and can provide early warning signs of environmental degradation or disease outbreaks that may also affect human health. LEAP is an additional method in the toolbox of conservationists, facilitating mortality assessments using a synergistic approach combining tracking data, site investigations, and necropsy analyses.

## Author Contributions

**Connor T. Panter:** formal analysis (equal), writing – original draft (lead), writing – review and editing (equal). **Carina Nebel:** writing – original draft (lead), writing – review and editing (equal). **Maximilian Raab:** data curation (equal), formal analysis (equal), investigation (equal), visualization (equal), writing – original draft (equal). **Verena Strauss:** conceptualization (equal), data curation (equal), investigation (equal), methodology (equal), writing – review and editing (equal). **Clara Freytag:** data curation (equal), formal analysis (equal), visualization (equal). **Manuel Wojta:** data curation (equal), formal analysis (equal). **Hannah Böing:** data curation (equal), formal analysis (equal). **Patrick Hacker:** data curation (equal), formal analysis (equal). **Rainhard Raab:** data curation (equal), formal analysis (equal). **Jendrik Windt:** data curation (equal), writing – review and editing (equal). **Annika Posautz:** investigation (equal), methodology (equal). **Anna Kuebber‐Heiss:** investigation (equal), methodology (equal). **Patrick Scherler:** data curation (equal), writing – review and editing (equal). **Martin U. Grüebler:** data curation (equal), writing – review and editing (equal). **Urs G. Kormann:** data curation (equal), writing – review and editing (equal). **Martin Kolbe:** data curation (equal), investigation (equal), writing – review and editing (equal). **Alexandre Millon:** data curation (equal), writing – review and editing (equal). **Javier de la Puente:** data curation (equal), writing – review and editing (equal). **Javier Viñuela:** data curation (equal), writing – review and editing (equal). **Duncan Orr‐Ewing:** data curation (equal), writing – review and editing (equal). **Oliver Krone:** data curation (equal), writing – review and editing (equal). **Torsten Langgemach:** data curation (equal), writing – review and editing (equal). **Susanne Åkesson:** data curation (equal), writing – review and editing (equal). **Brady Mattsson:** data curation (equal), investigation (equal), writing – review and editing (equal). **Petra Sumasgutner:** data curation (equal), writing – review and editing (equal). **Manuel Alcántara de la Fuente:** data curation (equal), writing – review and editing (equal). **Ernesto Alvarez:** data curation (equal), writing – review and editing (equal). **Juan Arizaga:** data curation (equal). **Albert Bach Pagès:** data curation (equal). **Ana Bermejo:** data curation (equal), writing – review and editing (equal). **Guido Ceccolini:** data curation (equal), writing – review and editing (equal). **Nayden Chakarov:** data curation (equal), writing – review and editing (equal). **Peter Derpmann‐Hagenström:** data curation (equal), writing – review and editing (equal). **Marek Dostál:** data curation (equal), writing – review and editing (equal). **Gerd Fabian:** data curation (equal), writing – review and editing (equal). **Wolfgang Fiedler:** data curation (equal), writing – review and editing (equal). **Manuel Galán:** data curation (equal), writing – review and editing (equal). **Clément Ganier:** data curation (equal), writing – review and editing (equal). **Andreas Gärtner:** data curation (equal), writing – review and editing (equal). **Liza Glesener:** data curation (equal), writing – review and editing (equal). **Alfonso Godino:** data curation (equal), writing – review and editing (equal). **Zuzana Guziová:** data curation (equal), writing – review and editing (equal). **László Haraszthy:** data curation (equal), writing – review and editing (equal). **Caka Karlsson:** data curation (equal), writing – review and editing (equal). **Katharina Klein:** data curation (equal), writing – review and editing (equal). **Ivan Literák:** data curation (equal), writing – review and editing (equal). **Nicolas Lorenzini:** data curation (equal), writing – review and editing (equal). **Manuela Löwold:** data curation (equal), writing – review and editing (equal). **Christopher Lüning:** data curation (equal), writing – review and editing (equal). **Boris Maderič:** data curation (equal), writing – review and editing (equal). **Karel Makoň:** data curation (equal), writing – review and editing (equal). **Kerstin Mammen:** data curation (equal), writing – review and editing (equal). **Ubbo Mammen:** data curation (equal), writing – review and editing (equal). **Torsten Marczak:** data curation (equal), writing – review and editing (equal). **Hynek Matušík:** data curation (equal), writing – review and editing (equal). **Aymeric Mionnet:** data curation (equal), writing – review and editing (equal). **Sara Morollón:** data curation (equal), writing – review and editing (equal). **Jakub Mráz:** data curation (equal), writing – review and editing (equal). **Winfried Nachtigall:** data curation (equal), writing – review and editing (equal). **Bernd Nicolai:** data curation (equal). **Marta Olalde Fernández:** data curation (equal), writing – review and editing (equal). **Meinolf Ottensmann:** data curation (equal), writing – review and editing (equal). **María Jesús Palacios González:** data curation (equal), writing – review and editing (equal). **Jean‐Yves Paquet:** data curation (equal), writing – review and editing (equal). **Vladimír Pečeňák:** data curation (equal), writing – review and editing (equal). **Lubomír Peške:** data curation (equal), writing – review and editing (equal). **Thomas Pfeiffer:** data curation (equal), writing – review and editing (equal). **Robert Pudwill:** data curation (equal), writing – review and editing (equal). **Dušan Rak:** data curation (equal), writing – review and editing (equal). **Tim Maximilian Rapp:** data curation (equal), writing – review and editing (equal). **Alexander Resetaritz:** data curation (equal), writing – review and editing (equal). **Stef van Rijn:** data curation (equal), writing – review and editing (equal). **Romain Riols:** data curation (equal), writing – review and editing (equal). **Arturo Rodríguez:** data curation (equal), writing – review and editing (equal). **Luisa Scholze:** data curation (equal), writing – review and editing (equal). **Laura Schulte:** data curation (equal), writing – review and editing (equal). **Aurélie de Seynes:** data curation (equal), writing – review and editing (equal). **Jan Škrábal:** data curation (equal), writing – review and editing (equal). **Péter Spakovszky:** data curation (equal), writing – review and editing (equal). **Eike Steinborn:** data curation (equal), writing – review and editing (equal). **Ján Svetlík:** data curation (equal), writing – review and editing (equal). **Samuel Talhoet:** data curation (equal), writing – review and editing (equal). **Miklós Vaczi:** data curation (equal), writing – review and editing (equal). **Anne‐Gaelle Verdier:** data curation (equal), writing – review and editing (equal). **Zdenĕk Vermouzek:** data curation (equal), writing – review and editing (equal). **Diego Villanúa Inglada:** data curation (equal), writing – review and editing (equal). **Jörg Westphal:** data curation (equal), writing – review and editing (equal). **Rainer Raab:** conceptualization (equal), data curation (equal), funding acquisition (equal), project administration (equal), supervision (equal), writing – review and editing (equal).

## Ethics Statement

Tagging and tracking of red kites was performed under permits issued by the relevant authorities in Austria (MIL2‐J‐0812/012, GFL2‐ J‐107/014, BHBRN‐2019‐314,986/5‐PS), the Czech Republic (S‐JMK 188552/2014 OŽP/Kuč, S‐JMK 32177/2015 OŽP/Kuč, S‐JMK 30634/2016 OŽP/ Ško, S‐JMK 177265/2017/OŽP/Ško), France (PP 987 A. Mionnet), Germany (BW: RPS35‐9185‐99/381, 55–7/8852.11; HE: RPKS‐23‐19 c 16/3–2020/1, JW‐1151; some of the tagging of juvenile red kites in HE took place within the framework of a cooperation under the animal testing permit number G 8/2018 of the Conservation Ecology Group at the Phillips‐Universität Marburg and under the species protection exemptions for ringing and extended tagging by the Helgoland Ringing Centre (Institute of Avian Research, IAR) and the State Ornithological Institute of Hesse; MV: 72213–2012/20, 661.35.5.2.000–02/22, VG‐22‐051, 44.30–2022‐148‐Gru, 66.1–55.40.30–1‐150; NI: H41L.22202/VB(H41L)_2022(Windt), 33.19–42,502–04‐21/3648; NRW: 81.02.04.2020.A188; ST: LAU 43.14–22,480‐75/2021, 43.13–22,480‐19/2018, 43.13–22,480‐21/2021 and 1/32 De), Luxembourg (N/Réf 90,832 CD/tw; N/Réf 93,179 CD/gp; N/Réf 95,445 CD/ne; N/Réf 102,316), the Netherlands (IvD‐light, April 23, 2009 (University of Groningen)), Portugal (875/2023/CAPT (ICNF)), Slovakia (MŽP SK 664/297/05–5.1pil and MŽP SK 2944/2017–6.3), Spain (INAGA 500201/24/2019/1147 & INAGA 500201/24/2019/11844 (Institute of Environmental Management of the Government of Aragon), EP/CYL/346/2019 & EP/CYL/66/2020 (Dirección General de Patrimonio y Política Forestal de la Junta de Castilla y León), 10/131599.9/19 & 10/168601.9/20 (Dirección General de Biodiversidad y Recursos Naturales de la Consejería de Medio Ambiente, Vivienda y Agricultura de la Comunidad de Madrid), VS/MLCE/avp_21_205 (Dirección General de Medio Natural y Biodiversidad), AUES/CYL/213/2021 & AUES/CYL/214/2022 (Dirección General de Patrimonio y Política Forestal de la Junta de Castilla y León), 10/126043.9/21 & 10/185882.9/22 (Dirección General de Biodiversidad y Recursos Naturales de la Consejería de Medio Ambiente, Comunidad de Madrid), CN0027/23/ACA—Dirección General de Sostenibilidad de la Junta de Extremadura y AMUS (Acción por el Mundo Salvaje), CN0045/24/ACA—AMUS (Acción por el Mundo Salvaje), CN0050/24/ACA—Dirección General de Sostenibilidad de la Junta de Extremadura), and Sweden (ethical permit from Malmö‐Lunds Djurförsöksetiska Nämnd 5.8.18–06518/2020, M74‐20; Ringers License no. 440 to SÅ from the Swedish Environmental Protection Agency and the Swedish Ringing Office).

We performed all methods following the relevant guidelines and regulations concerning study animals. We confirm that the study is reported in accordance with ARRIVE guidelines (https://arriveguidelines.org/).

## Conflicts of Interest

This publication was produced as part of the LIFE EUROKITE project, which is funded by the European Commission's LIFE Nature program (60%), grid operators (15.8%), nature conservation NGOs (9.2%), authorities (8.8%) and renewable energy companies (6.2%). The LIFE EUROKITE project is based on an international cooperation of scientists, NGOs, governmental authorities, and companies, representing diverse backgrounds and contributing to this study in different ways. Based on the legal setup of the LIFE project, co‐financers only provide funding but have no say in the content of scientific contributions. Prof. Dr. Martin Bergmann, who works for iTerra energy GmbH, conducted a mandated quality assurance review to ensure scientific standards. Financial contributors from private companies and NGOs were largely responsible for tagging birds and providing data to the project, while data evaluation, interpretation, and writing of the manuscript were performed by authors working for independent research institutions and universities.

## Supporting information




Appendix S1.

Appendix S2.

Appendix S3.

## Figures and Tables

**FIGURE B1:**
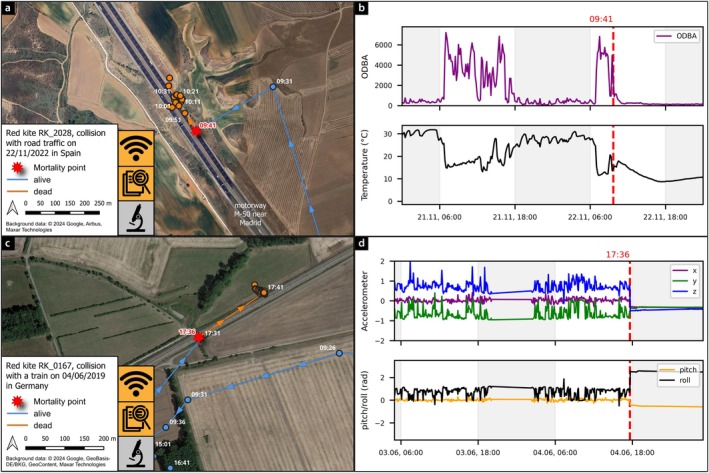
Exemplary case studies of mortality analyses from tracking data of two red kites (*Milvus milvus*) of the LIFE EUROKITE project. The maps show the GPS locations before death (blue—alive; orange—dead), with points being connected with arrows to depict movement. The location of mortality is shown with a red star (“mortality point”). The icons in the bottom left box symbolize the availability of the information sources including: Tracking data (Wi‐Fi symbol), site investigation (documents with magnifying glass) and necropsy (microscope; orange—yes; gray—no): (a) tracking of RK_2028's collision with a vehicle, (b) metadata of the interrex transmitter, showing overall dynamic body acceleration (ODBA), a sum of parameters from the accelerometer data, and temperature. (c) Tracking RK_0167's collision with a train, (d) accelerometer (top) and pitch/roll data (bottom). All times are given in UTC.

**FIGURE B2:**
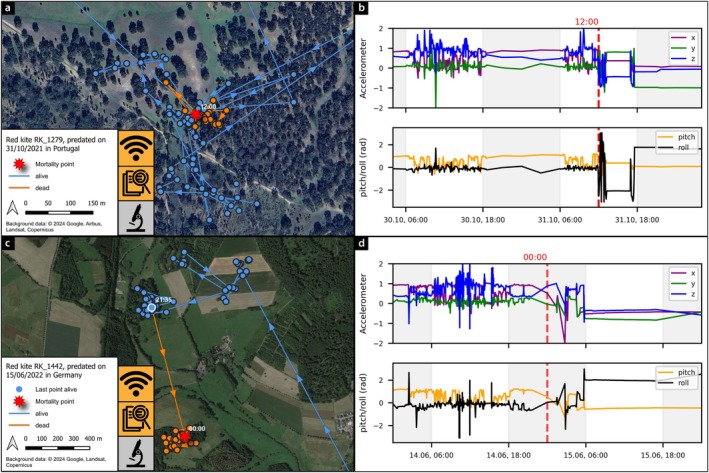
Exemplary cases of mortality analyses from tracking data of two red kites (*Milvus milvus*) from the LIFE EUROKITE project that died through predation. The maps show the GPS locations before death (blue—alive; orange—dead), with points being connected with arrows to depict movement (blue—alive, orange—movements of the carcass by the predator or scavengers after death. The mortality location is shown with a red star (“mortality point”). The icons in the bottom‐left box indicate information sources for mortality assessment: Tracking data (wi‐fi symbol), site investigation (documents with magnifying glass) and necropsy (microscope; orange—yes; gray—no): (a) tracking of RK_1279, (b) Transmitter metadata of RK_1279: Top plots show accelerometer data, bottom plots the pitch/roll data. The moment of mortality is marked with a red, dashed vertical line. (c) tracking of RK_1442, (d) transmitter metadata of RK_1442. All times are given in UTC.

**FIGURE B3:**
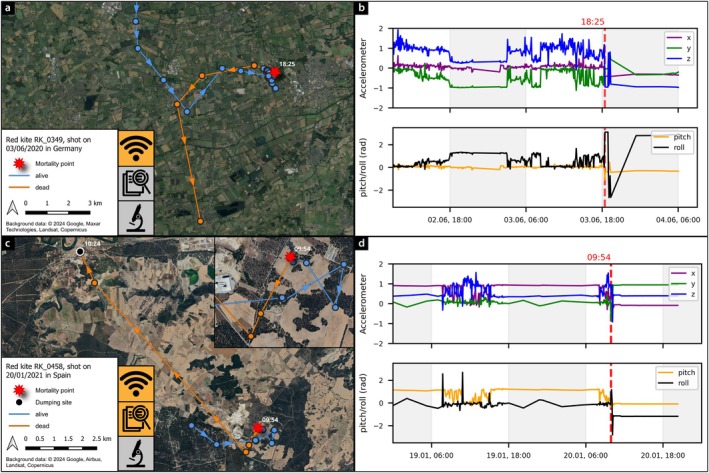
Exemplary case studies of mortality analyses from tracking data for two red kites (*Milvus milvus*) from the LIFE EUROKITE project that died by illegal shooting. The maps show the GPS locations before mortality occurred (blue—alive; orange—dead), with points being connected with arrows to depict movement (blue—alive, orange—movements after death) of the carcass by the predator or scavengers. The mortality location is shown with a red star (“mortality point”). The icons symbolize the availability of the information sources including: tracking data (Wi‐Fi symbol), site investigation (documents with magnifying glass) and necropsy (microscope; orange—yes; gray—no): (a) tracking of RK_0349, (b) transmitter metadata of RK_0349: Top plots show accelerometer, bottom plots the pitch/roll data. The moment of mortality is marked with a red, dashed vertical line. (c) Tracking of RK_0458, (d) transmitter metadata of RK_0458, same as (b). All times are given in UTC.

**FIGURE B4:**
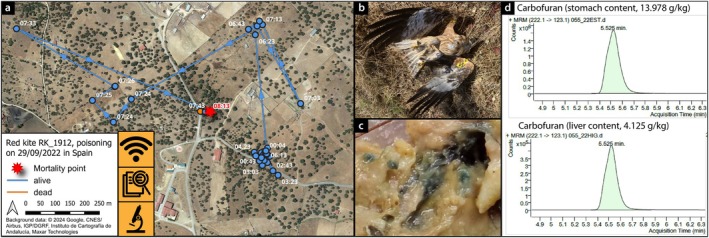
Exemplary case study of poisoning victim RK_1912: (a) tracking locations of RK_1912 (blue—alive; orange—dead), with points being connected with arrows to depict movement. The mortality location is shown with a red star (“mortality point”). Time of some GPS locations are shown. The icons symbolize the availability of the information sources including tracking data, site investigation and necropsy (orange—yes; gray—no). (b) Photo of the carcass as found in the field; (c) black microgranules suggesting Aldicarb in the digestive contents. (d) High‐performance liquid chromatography coupled with mass spectrometry (HPLC‐MS/MS) chromatograms identifying carbofuran. All times are given in UTC.

**FIGURE B5:**
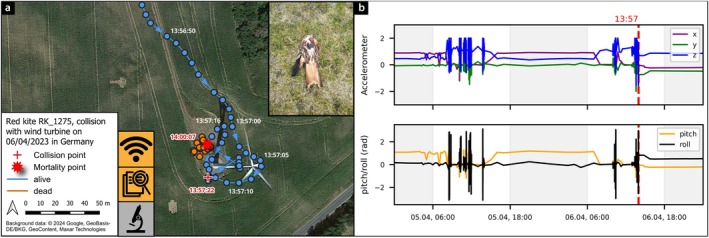
Exemplary case study of wind turbine collision victim RK_1275: (a) tracking locations (one‐data‐point‐per‐second resolution, blue—alive; orange—dead), with points being connected with arrows to depict movement. The mortality location is shown with a red star (“mortality point”). Time of representative GPS locations are shown. The icons symbolize the availability of the information sources including tracking data, site investigation and necropsy (orange—yes; gray—no). The inset shows the carcass of the kite as found by site investigators at the mortality location; (b) accelerometer (top) and pitch/roll data (bottom). All times are given in UTC.

## Data Availability

The data used for producing the results is available at https://figshare.com/s/9749e4adf137bf16ebd8.

## Appendix A

Glossary showing key terms used throughout the LIFE EUROKITE Assessment Protocol with definitions.

**Table:**

Terms	Definition
Bird crime	Illegal trapping, shooting and poisoning of birds. In Europe, unintentional poisoning of many protected species is illegal (e.g., the red kite), and thus represents a crime. Furthermore, illegal substances might be responsible for the death of a kite (e.g., carbofuran has been banned in the EU).
Carcass retrieval location	The location where the carcass was retrieved. Note that this location may not be the same as the mortality location for several reasons detailed below.
Case Form	A form that allows standardized data collection during a site investigation. It gives guidance to be aware of relevant information that needs to be collected. The LEAP Case Form is available as Supporting Information S2.
Cause of mortality	Specific reason that led to an individual's death and its identification is the aim of the investigation. Here, we only consider the primary cause of death, which directly resulted in mortality. Information on secondary causes should be documented if available.
Local partners	Network of collaborators that covers a large spatial extent throughout the distribution range or study area of the species of interest. A large network of local partners is an integral part of LEAP to ensure rapid carcass retrieval.
Moment of death	The moment when the tagged animal's vital functions cease, and it is dead. Here, the moment of death is based on tracking data, defined as the first timestamp the bird is detected as dead. The time can be determined based on accelerometer or pitch/roll data, but also an immobile location and declining temperature data. If the tag stops working while deployed, the case is ruled as “tag failure” and not considered as mortality.
Mortality location	Location of mortality derived using GPS locations at the moment of death. It can be characterized by a succession of consecutive locations in the same place without displacements and typical transmitter metadata described below. The mortality location may differ from the later retrieval location. In cases where the bird was found after the tag stops working, the retrieval location was assumed as mortality location unless the site investigation shows otherwise, e.g., a plucking place is found.
Necropsy	Examination of an animal carcass after death by veterinary pathologists or trained veterinarians. The aim of a necropsy is to determine the cause of mortality or extent of disease, and often involves dissection, micro‐ and macroscopical observations, further analyses such as histology, microbiology or toxicology and finally interpretation and documentation. During the necropsy, a Necropsy Form should be filled out.
Necropsy Form	A standardized form that details the necropsy and laboratory procedures carried out and concludes with the most likely cause of mortality. The LEAP Necropsy Form is given as Supporting Information S3.
Opportunistically collected carcasses	Carcasses collected outside of a specific protocol. Most studies assessing mortality causes in wild populations use carcasses found by chance or systematic searches of certain areas in the wild. Such carcasses are often in bad condition due to the prolonged time between the moment of death and the retrieval of the carcass, limiting assessments of the cause of mortality. Furthermore, the chances of finding such carcasses are dependent on the reason of death (e.g., higher for a roadkill/wind turbines collision victim in contrast to a botulism/predation victim that dies in a remote area). Biases associated with data obtained from such collections are therefore inevitable.
Principal investigator	Person responsible for managing the project, preferably an expert of the relevant species and recognized by all involved local partners and authorities.
Site investigation	Process of searching for carcasses and retrieval by one or more site investigators. Site investigation involves both visiting and investigating the mortality location and the carcass retrieval location.
Site investigator	Person(s) responsible for carcass retrieval and investigation of the mortality and carcass retrieval locations, appointed by local partners. The site investigator is also responsible for filling out the Case Form and returning it to the tracking data coordinator. It is recommended that inexperienced site investigators are supported by experienced investigators either on‐site or available via stand‐by to ensure replicable and thorough site investigations. The guideline for site investigators is presented as Supporting Information S1.
Tracking data	Modern GPS transmitters supply various metadata, including the GPS location (longitude, latitude), additional movement sensor data (three‐dimensional accelerometer data from the transmitter in the form of “x,” “y,” and “z” variables, which describe the direction vector of the transmitter, and pitch/roll data, which describes the angle of movement). The accelerometer data can also be summarized, e.g. into overall dynamic body acceleration (ODBA). Furthermore, transmitters may provide internal information (battery charge) and external variables (temperature, air pressure). Modern transmitters can also include barometric data, which allows precise calculations of flying height through air pressure. Note that the type of data collected by transmitters varies between manufacturers and models, but modern versions of > 5 g commonly deliver this kind of data.
Tracking data coordinator	Person(s) responsible for monitoring the tracking data, database management and administration, and communicating with on‐the‐ground site investigators.

## Appendix B

### Applications of the LEAP framework with examples

Here, we present eight individual cases of red kite mortality events and demonstrate how LEAP was used in each case to determine the cause of mortality. We provide examples from a “certain” road traffic collision (B.1) and a “certain” collision with a train (B.2). We also highlight two cases of predation, as the LEAP approach can help detect predation and distinguish this from scavenging based on information from tracking and site investigation (B.3). We present two illegal shooting events where tracking data alone allowed a “certain” determination of the cause of death and one “certain” case of poisoning (B.4). We also provide a case of “certain” wind‐turbine collision based on GPS tracking at one data‐point‐per‐second resolution (B.5).

### Ruling out causes of mortality early

Infrastructure‐related deaths can be excluded if no anthropogenic structures, such as wind turbines, train tracks, roads, or power lines, are present in the area surrounding the mortality location. If the carcass is recovered whole and with no signs of animal contact, predation can be ruled out even before a necropsy.

#### B.1. Certain road collision of RK_2028 tagged as an immature bird in 2022

The first case focuses on red kite RK_2028, which died on November 22, 2022, on the M50 highway northeast of Madrid (Figure B1a,b). The kite spent its final moments near the road, with the tracking data indicating an attempt to cross the highway. The tracking data then indicated a collision and subsequent movement along the road followed by stable pitch/roll by the side of the highway. This unnatural movement immediately after total stasis following the time of death led to the “certain” conclusion that the bird died from a collision with a vehicle.

#### B.2. Certain train collision of RK_0167 tagged as a fledgling in 2018

The bird died on June 4, 2019, in Germany. The tracking data and metadata showed that the bird experienced an impact and was dragged along the railway line, where it sent subsequent GPS locations 5 min post‐collision (Figure B1c,d). The bird remained there and was later moved away from the train tracks, either by a scavenger or a human. Due to the proximity to the train tracks, clear signs of collision, and dragging along the tracks immediately after the time of death, the cause of mortality was classified as a “certain” collision with a train.

#### B.3.a Certain predation of RK_1279 tagged as a fledgling in 2021

Red kite RK_1279 (Figure B2a,b) died in Portugal on October 31, 2021. Around 12:00, the transmitter reported a sharp spike in pitch/roll and accelerometer data, which indicated the moment of death. Immediately after death, shaking of the carcass was evident by strong oscillations in the pitch/roll data. Such oscillations indicate probable plucking or feeding on the carcass. Around 17:00, new movement of the carcass was recorded, and the accelerometer data indicated that the carcass was flipped over, likely due to scavenging activities. Only the GPS transmitter and some bones, including a foot, were recovered by site investigators on November 5, 2021.

Feeding on the carcass produces clear patterns in the accelerometer data that do not reflect natural movements of living birds. While the accelerometer data and pitch/roll data show typical movement patterns before death (the moment of death is indicated by the red line, Figure B2a,b), the transmitter and body orientation change immediately when the kite was killed. Due to the clear pattern in the accelerometer data that indicates feeding on the carcass immediately after death, this case was ruled as “certain” predation followed by scavenging. The mortality cause would have been inconclusive in the absence of tracking data, because we would not be able to differentiate predation from any other cause.

#### B.3.b Probable predation of RK_1442 tagged as a juvenile in 2021

Red kite RK_1442 died on June 15, 2022, in Germany. Between June 14 21:35 (UTC) and June 15 00:09, a data gap occurred when the data frequency was reduced to save energy during the night. The kite was alive at 21:35, died by 00:09, and was moved to a different location. The data suggested that the bird died at night via predation by a nocturnal predator sometime between those time periods. Using the accelerometer and temperature data, the death of the bird was confirmed. During the following night, GPS locations showed that the carcass was moved to an area 1.89 km east of the mortality location. There, a red kite foot and feathers were found near a Eurasian eagle owl (*Bubo bubo*) nest. Eagle owls are intra‐guild predators of diurnal raptors that rarely practice scavenging (e.g., Fernández‐García et al. 2024).

Often, the time of day and the time between death and data indicating consumption of the carcass are essential to differentiate between predation and scavenging. The time between death and feeding initiation can be difficult to judge, especially when data are transmitted at irregular intervals, but feeding on the carcass will usually occur shortly after the kill. During the day, data intervals are typically set to one data point every 5 min. Given this information, we classified the case as a “probable” predation event, likely by an eagle owl. The case was classified as probable to account for the (unlikely) possibility of scavenging by the owl.

#### B.4.a Certain illegal killing of RK_0349 as an immature bird in 2020

Red kite RK_0349 was shot in Germany on June 3, 2020 (Figure B3a). The bird moved following typical flight patterns over fields and forests before a sudden spike in accelerometer data and roll/pitch at 18:25 (Figure B3b). The GPS locations remained the same until approximately 18:50, when the transmitter recorded movement toward the south along the road. Within 25 min, the kite had moved 12.4 km to a nearby village. Given the distance covered within this short period and the tracking data indicating that the kite “used” roadways, we suspected that the carcass was transported in a vehicle. The GPS transmitter continued to provide data until 06:02 the next morning. The transmitter was moved (potentially without the carcass) to a location that prevented the battery from charging. The last transmission was obtained at 19:15. Despite no carcass being retrieved and LIFE EUROKITE not being contacted by the retriever of the GPS tag, there can be no doubt that the illegal shooting of that individual followed by transport in a car occurred, resulting in a “certain” illegal shooting classification.

#### B.4.b Certain illegal killing of RK_0458 tagged as a fledgling in 2020

Red kite RK_0458 migrated to Spain, where it overwintered and subsequently died on January 20, 2021 (Figure B3c,d). In the morning, the bird rested near an open field. It flew over the field at 09:24 and was likely shot at 09:34 over the same field. At 10:04, the transmitter was moved along the roadways, away from this last location to a nearby village. There, the transmitter sent the same GPS locations from 10:24 onward and was found at 20:18 in the garbage by site investigators who were alerted to the bird's death immediately by the tracking data coordinator. The transmitter webbing had been cut off and disposed of, with the carcass of the bird never being recovered. Due to the transmitter webbing showing clear cut marks and the movement data recorded by the transmitter at the time of death and shortly thereafter, we classified this as a “certain” case of illegal shooting.

#### B.4.c Certain illegal killing of RK_1912 as a juvenile in 2022

Red kite RK_1912 died in Cerro de la Mina in the Spanish province Badajoz on September 29, 2022, and its carcass was found there on September 30 (Figure B4a). Observations during site investigations (Figure B4b) suggested poisoning as a potential cause of death as the carcass was in perfect state without any signs of trauma or predation, and therefore the police were informed. The carcass was sent to the Veterinary University of Extremadura's toxicology laboratory, where pathological examination revealed the presence of black microgranules in the digestive contents, suspected to be Aldicarb (Figure B4c).

Using samples of the bird's digestive contents and liver, carbofuran poisoning was identified by toxicology (Figure B4d), using high‐performance liquid chromatography coupled with mass spectrometry (HPLC‐MS/MS) and gas chromatography coupled with mass spectrometry (GC–MS/MS). Due to the definite result of the necropsy, with the available tracking data and site investigation information indicating no contrary information, the case was ruled as “certain” poisoning by carbofuran.

### B.5 Certain wind turbine collision of RK_1275 tagged as a fledgling in 2021

Red kite RK_1275 died on April 6, 2023, and was found below a wind turbine. Due to the high spatial precision and temporal resolution of the GPS location data, that is, one data point per second (Figure B5a), the exact movements of the bird unambiguously revealed its collision with the wind turbine. Furthermore, the data showed that the bird might have survived the initial collision but was able to move a short distance before the moment of death (Figure B5a,b). Following detection and a search by the LIFE EUROKITE team, the carcass was found in a fresh state within turbine proximity. Despite only two of the three information sources available (no necropsy has been performed yet), we classified this case as “certain”

## Appendix C

### Examples of evidence to support certainty categories

#### C.1 Certain mortality assessment with all three data sources

Poisoning: Clear signs of poisoning on the carcass or in the surrounding area (e.g., a poison bait is found), making poisoning a certain mortality cause.

Wind turbine collision: severe mutilations, for example, being cut in half, indicate death through collision with a turbine. Bloodstains from the collision being visible on rotor blades above the carcass are also clear signs of a collision. An alternative proof of a collision is the set of transmitter locations and associated metadata with high temporal resolution (i.e., one‐second data, see example of a collision victim in B.5).

Electrocution: visible on the carcass in the field by clear burn marks on the feathers or talons, and sometimes also the bill. Smaller burn marks emit an odor and can be smelled more easily than seen. It is important to differentiate between electrocution on power lines and collisions with power lines. The latter will not show any of the clear signs of electrocution mentioned here, but carcasses will be found in proximity to power lines.

Predation: Carcass remains are found in nests of non‐scavenging species like northern goshawk (*Accipiter gentilis*), as well as plucking sites.

**TABLE C1:** Causes of mortality for the red kite (*Milvus milvus*), separated into subcategory, main category and summary category, as used for LEAP for red kites in this study, as example of different mortality factors for an investigated species. Certainty factors are chosen based on main categories.

Summary	Main category	Subcategory
Anthropogenic	Poisoning	Anticogulants/Coumarin derivatives
		Cholinesterase inhibitors (Carbofuran)
		Lead
		Other substances
		Landfill Pentobarbital
	Road traffic	Collision
		Road traffic—collision with car
	Power line	Electrocution
		Electrocution on distribution pole
		Electrocution on Interchange/transformer
		Electrocution on train power lines
		Collision
		Collision with cable
		Collision with tower
	Shooting/Trapping	Shooting
		Trapping
	Rail traffic	Collision
	Wind turbine collision	Rotor blades
		Tower
	Drowning	In water tank
		In slurry tank
		Others
	Anthropogenic (other)	Collision with airplane
		Agricultural machine
		Tree was cut down
		Fixation of logger
		Traceable human intervention
		Trauma
Natural	Predation	Goshawk
		Eagle owl
Natural	Predation
		Racoon
		Eagle
		Hawk
		Fox
	Other
	Disease	Mixed infection
		Parasitic infection
		Bacterial infection
		Viral infection
		Mycotic infection
	Trauma (natural)	Fallen out of nest
		Tree broken
		Destruction of aerie, e.g., by wind
		Trauma—fight with another raptor
	Starvation	Starvation
	Natural (other)	Fledgling accident
Cainism	
Inconclusive	Inconclusive	Unknown trauma
		Inconclusive

**TABLE C2:** Country distribution of LEAP mortality cases collected from 2019–2022 based on availability of necropsy (TSN with necropsy vs. TS).

Country of death	TS	TSN	LEAP‐pooled
Austria	2	9	11
Belgium	3	1	4
Czech Republic	17	8	25
Denmark	2	0	2
France	24	30	54
Germany	77	19	96
Greece	0	1	1
Hungary	1	0	1
Italy	6	2	8
Poland	3	0	3
Portugal	6	4	10
Serbia	1	0	1
Slovakia	3	4	7
Slovenia	1	0	1
Spain	74	25	99
Sweden	1	0	1
Switzerland	3	1	4
Tunisia	1	0	1
**Total**	**225**	**104**	**329**

**TABLE C3:** Species distribution of opportunistically retrieved carcasses.

English name (scientific name)	Count
Barn Owl ( *Tyto alba* )	1
Bearded Vulture ( *Gypaetus barbatus* )	1
Common Buzzard ( *Buteo buteo* )	45
Common Kestrel ( *Falco tinnunculus* )	2
Eastern Imperial Eagle ( *Aquila heliaca* )	3
Eurasian Eagle‐Owl ( *Bubo bubo* )	2
Eurasian Sparrowhawk ( *Accipiter nisus* )	1
Golden Eagle ( *Aquila chrysaetos* )	2
Northern Goshawk ( *Accipiter gentilis* )	3
Peregrine Falcon ( *Falco peregrinus* )	1
Red Kite ( *Milvus milvus* )	7
Saker Falcon ( *Falco cherrug* )	2
Tawny Owl ( *Strix aluco* )	3
Ural Owl ( *Strix uralensis* )	5
Western Marsh Harrier ( *Circus aeruginosus* )	53
White‐tailed Eagle ( *Haliaeetus albicilla* )	12
Unknown species (N/A)	2

**TABLE C4:** Details on carcass state categories and examples of two carcasses for each. Better resolution images can be found in the supporting information.

**Very good** Carcass intact Sometimes still warmer to the touch compared to ambient temperature Rigor mortis starting or in progress No insects on the body		
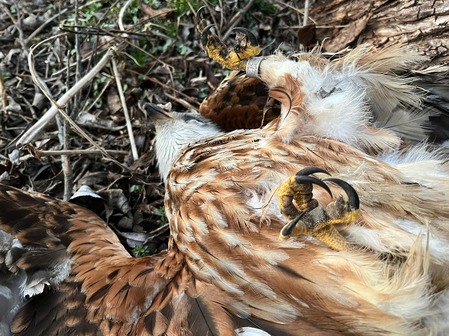
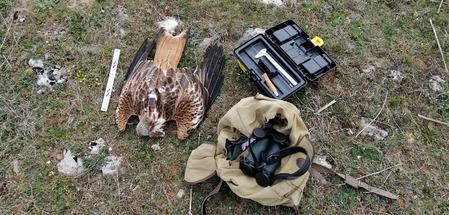
**Good** Rigor mortis completed before discovery No insects on the body Fly eggs may be present around the eyes or cloaca Little to no decomposition If the animal's body is dismembered: organs are fresh, firm and moist		
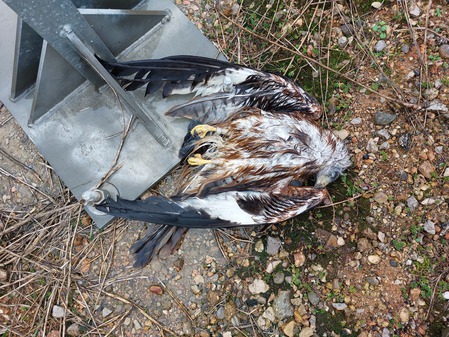
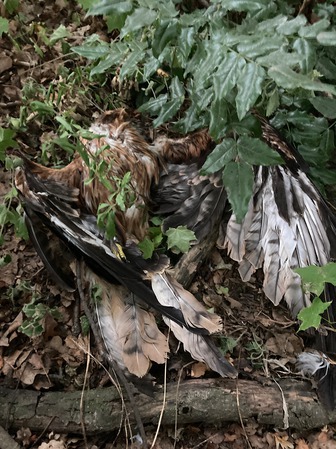
**Medium** Odor may be present, beginning decomposition Eyes are sunken but clearly visible Fly larvae and insects may be present If the animal's body is dismembered: organs are starting to become mushy/dry on the surface		
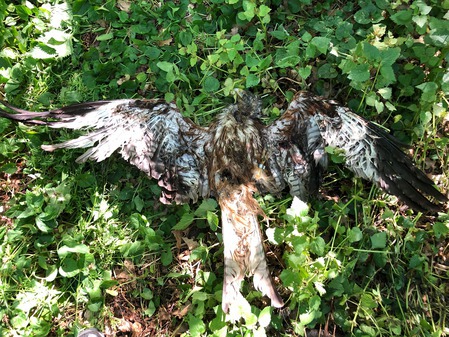
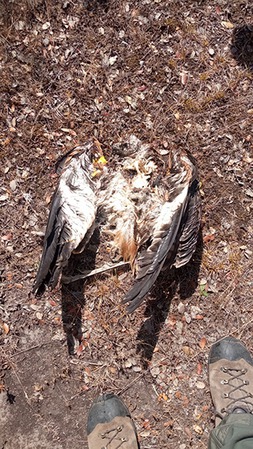
**Bad** Advanced decomposition Eyes are sunken but clearly visible Beetles are predominant on the carcass If the animal's body is dismembered: organs are mushy and liquefying		
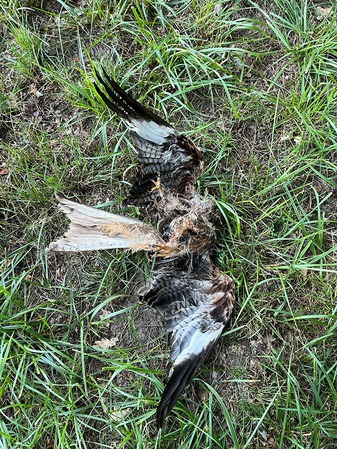
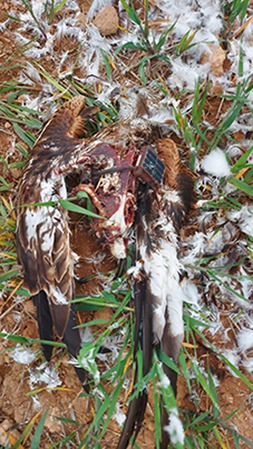
**Very bad** Eyes are sunken and barely visible The body is completely dry Very light in weight Bones and feather remnants present Partial disarticulation of individual bones		
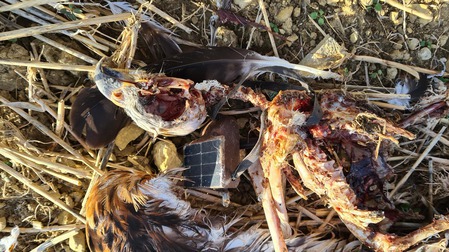
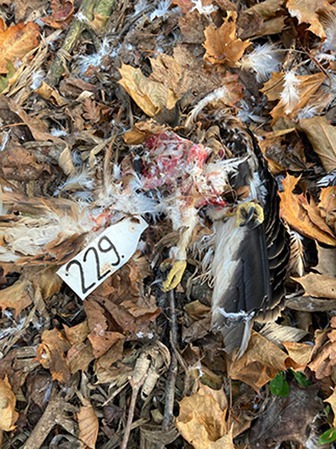

#### C.2 Certain mortality assessment without a carcass

Shooting/Predation: The transmitter metadata provides evidence of the transmitter being moved immediately after the death of the bird, which is suspicious and indicates either illegal shooting or predation. If strong oscillations in the pitch and roll data are detected immediately after the moment of death, this suggests feeding or plucking in line with predation (B.3.a). If, after death, the carcass moves along roadways at unnatural speeds and is subsequently discarded in human areas, this certainly represents an illegal shooting event, with the carcass removed by the perpetrator. Examples of this are shown in B.4.

Poisoning: When a GPS transmitter is retrieved and evidence suggests that the Teflon harness has been cut, human involvement and potential illegal activities are certain. Tracking data allows distinction between illegal shooting and poisoning in such cases based on the movement and metadata of the GPS locations, for example, transport on the road immediately after death indicates shooting, whereas no movement after death indicates poisoning. See examples in B.4.

#### C.3 Probable mortality assessments

Train collision: A red kite is found dead near a train track. The carcass is in bad condition and necropsy examination is not possible. Due to the proximity to the track, the most likely a priori causes of death include collision with a train, collision with an overhead power line, or electrocution on an overhead power line. Disease, poisoning, or predation are also possible. The site investigation reveals that there is no overhead power line, ruling out collision with it or electrocution. Tracking data shows typical signs of a collision, one sharp peak in accelerometer and pitch/roll followed by no movement and staying in an unnatural orientation. Predation is ruled out due to there being no indication of feeding off the carcass after the mortality event based on pitch/roll data. Disease is ruled out by looking at the GPS tracking of the last days, where no abnormalities that could result from disease or poison effects are observed. By combining all the available information from site investigations and tracking data, a train collision is the only plausible cause of mortality.

Wind turbine collision: A red kite is determined to have died close to the wind turbine based on GPS tracking data. The tracking information also shows clear signs of a collision (i.e., spike in pitch/roll) followed by a sudden lack of movement. The bird and tag are then moved, presumably by a scavenger, across the field surrounding the turbine to their final resting place along the forest edge. The carcass of the bird is not found at the search, nor are there any signs of the bird or mortality causes other than the wind turbine, so no necropsy is possible. As no blood on the rotor blades of checked turbines was observed and there are also no high‐resolution tracking data available, a certain evaluation in this case is impossible, as the definitive proof is lacking, but all indications point toward a collision and no other cause of death.

Poisoning: There was a poisoned bait discovered ca. 50 m from the last transmitter location, and the bird was moving less during its last days of life. However, the tracking data never showed direct overlap of bird movement and bait location in this case. As no carcass was found, without a necropsy it remains uncertain whether the kite actually ate the bait, but its behavior points to probable poisoning.

#### C.4 Examples of evaluation as possible mortality

Road collision: A bird flies until it crosses a public road, and afterward, no tracking data is available, indicating the potential destruction of the transmitter by a collision. Due to the transmitter likely being destroyed by the collision, which likely also killed the bird, no detailed information about the death and the time after is available, and the carcass could not be retrieved. As information is limited to tracking data, other reasons, such as failure of the tag with the bird being alive or predation with immediate movement of the carcass into a hole or shooting along the road, cannot be excluded for certain. Shooting along a public road is also less likely than a collision, considering the risk of being caught in an illegal act. The available information therefore points to a collision with a vehicle as the most likely cause of death.

Power line collision/electrocution: The bird is found with its intact transmitter directly under a power line, but the condition of the carcass is insufficient to ascertain power line‐related wounds, and no necropsy can be performed. Although the position of the bird under the power line indicates an electrocution or collision event, there are other causes of death (e.g., predation or shooting) that cannot be fully ruled out. In this case, the transmitter did not collect a sufficient amount of metadata during the minutes leading up to the death due to low‐light conditions and therefore cannot provide support for or against other potential causes of death.

Predation: The bird died during sunset with signs of being healthy beforehand based on tracking data. The carcass is not suitable for necropsy and shows signs of predation (e.g., plucked feathers), but scavenging following another mortality cause (e.g., shooting, poisoning or a fight with another raptor) cannot be excluded because the transmitter was low on battery and only sent infrequent data after the moment of death. The location after death is located away from the roosting site, indicating that the carcass was moved after the bird being killed at its roosting site. GPS tracking data did not show any signs of disease, for example, reduced movement in the days before death. Therefore, a possible predation classification is assigned to this case.
