# Supplementary material for: A LEAP Forward in Wildlife Conservation: A Standardized Framework to Determine Mortality Causes in Large GPS‐Tagged Birds
Source: Ecol Evol. 2025 Mar 27;15(4):e70975. doi: 10.1002/ece3.70975 (PMC11949540; doi:10.1002/ece3.70975)
Supplement: Supplementary file 1 — Appendix S1. [file ECE3-15-e70975-s002.pdf]

LIFE EUROKITE Assessment Protocol: Case Form

CASE CODE

___ - ___ - ___ - ___ - ___

DateOfSearch(YYYYMMDD) – CountryCode – ZipCode – SatelliteTagCode – NumberOfCaseForm

SENDER / FINDER

Date of completing Case Form __ / __ /20__

Search alert by (Name/Institution) ___ Date of Search __ / __ /20__

Number of involved searchers ___ Dog included in search ☐ Yes ☐ No

Name / e-mail address / phone number of the searcher:

Additional notes on the finder and the course of discovery (e.g. alerted searcher, chance find by hunter or pedestrian):

Travel time: __ h __ min (Start __ : __ / Ending __ : __)

Distance covered: __ km (Start __ km / Ending __ km)

Search time: __ h __ min (Start __ : __ / Ending __ : __)

Searched area size: __ m2

Additional notes:

SATELLITE TRANSMITTER / BIRD HAS BEEN FOUND

GPS-transmitter found: ☐ Yes ☐ No Satellite tag code: ___

Last date of sending information/time of death according to transmitter: __ / __ /20__

Carcass found: ☐ Yes ☐ No Species: ___

Additional notes (e.g. abnormalities, indentification markings/ring number, sex, age)**:**

LOCALITY DETAILS

Date and time of finding: __ / __ /20__ __ h __ min

Take photos and attach them: ☐ Overview ☐ Makro ☐ Surrounding

Location of carcass (GPS-coordinates): ___

Region and Country: ___

Radius of searched surrounding: ___ m

Surroundings/Vegetation:

☐ Streets (__ m) ☐ Train tracks (__ m) ☐ Forest/trees/breeding sites (__ m)

☐ Power lines (__ m) ☐ Agricultural land (__ m) ☐ Wind turbines (__ m)

Further information and additional notes like weather conditions, hunting management plan, predator-control authorisation, name of company, ID of pylon / windturbine / street ect.:

EVALUATION OF CASE

Case suspicious? ☐ Yes ☐ No

Suspected cause of death:

- More precise cause of death: Trauma
  More precise cause of death: Drowning

Additional notes on suspected cause of death (e.g. possible predator, more precise cause of death,...):

Police informed? ☐ Yes ☐ No Whom:

Police reference number:

Authority informed? ☐ Yes ☐ No Whom:

Others (Name, Department) :

Additional notes:

EVIDENCES COLLECTED

Samples collected (including carcass as sample)? ☐ Yes ☐ No Total number of samples:

Collected by:

Short description of samples:

Further investigation of samples: ☐ Yes ☐ No By (Laboratory):

Transport carried out by: Date: / /20

Carcass remains with searcher: ☐ Yes ☐ No Done by:

Additional notes:

ADDITIONAL INFORMATION CONSIDERING CASE FORM

Time necessary to complete form: min

Additional notes on form completion (questions, improvements, ect.):

→ REFRIGERATE carcass if it can be submitted*
directly to pathology lab for necropsy
(transport time not more than 1-2 days)

→ FREEZE carcass if it will not be submitted*
directly to pathology lab
(transport time more than 1-2 days)

*Use specialized courier services only.

Thank you for your cooperation!
