# Supplementary material for: A LEAP Forward in Wildlife Conservation: A Standardized Framework to Determine Mortality Causes in Large GPS‐Tagged Birds
Source: Ecol Evol. 2025 Mar 27;15(4):e70975. doi: 10.1002/ece3.70975 (PMC11949540; doi:10.1002/ece3.70975)
Supplement: Supplementary file 2 — Appendix S2. [file ECE3-15-e70975-s001.pdf]

LIFE EUROKITE Assessment Protocol: Mortality Protocol

This protocol was developed to provide guidance for people actively involved in the search of dead birds within the LIFE EUROKITE Project.

To determine the influences of anthropogenic factors and the key reasons of mortality of Red Kite populations, more than 1.000 Red Kites and several other birds of prey were equipped with GPS/GMS transmitters within the LIFE EUROKITE project. Additionally, several hundred tagged Red Kites are shared with the LIFE EUROKITE project from partners and cooperation partners. In particular, information on causes of mortality can lead to very important hints, which highlight the main influences the populations are struggling with. Especially bird crime and human-caused mortality reasons are very topical factors, and several studies have shown that illegal persecution is one of the most influential factors in decreasing bird populations, along with habitat reduction.

This mortality protocol has its aim in providing guidance on what to do in case a dead bird is found, either using satellite tags or randomly from hunters, farmers or pedestrians.

The following plan focuses on the Red Kites that have been tagged as part of the LIFE EUROKITE project, but can be transferred in all cases, where a dead bird (especially birds of prey) has been found and the cause of death may be suspect.

<u>Preface</u>

The transmitter signals of the tagged birds are checked up to three times a day with regard of i) transmitter frequency and area, where the signal comes from, ii) position of the transmitter and iii) temperature.

If the transmitter

- sends a **signal from the same area** for more than 24 h
- and/or the **position of the transmitter** remains more than 24 h the same
- and/or the **temperature decreases** for more than 24 h

the bird is graded as “suspected to be dead” and the attached flow chart helps with a cascade of actions to deal with further steps.

If at least one of the three listed options may be the case, there are two possible circumstances:

- Satellite tag detached from the bird
- Tagged bird is suspected to be injured or dead

The responsible person of the concerning country should be informed to take further action, including the search for the alleged dead bird.

LIFE EUROKITE Assessment Protocol: Mortality Protocol

The Search

The search will start at the point, where the last signal of the satellite transmitter has been. Data for the search are provided by the project. Please note, that data which were made available to you for the search of dead birds are not allowed to give to third parties without our confirmation. Depending on the GPS signal it might be that the last position of the satellite tag is not 100 % correct, so please make sure to search also in the surrounding. Sometimes satellite tags can hang in the trees etc. so please be aware of that and check the tress etc. around. If the satellite transmitter and/or the bird has been found do not touch it and follow the next steps:

If only the satellite tag has been found a visual inspection of the terrain, at least a minimum of 100m radius, including a systematic ground search of the surrounding area should be carried out to look for evidences.

→ **Take photos** of the area and the bird in all cases.

→ **Always keep contact with the LIFE EUROKITE team** (the person who contacted you) before doing anything with the bird/satellite tag

→ **Contact local and regional/national police** and **report circumstances** in cases of suspicion of illegal activities. **Please wait for police and do not remove carcass.**

→ **Circumstances should be investigated regarding to illegal persecution and suspected bird crime** (see below). This step should be **done by police**, may **with the help of specialists**.

→ **Case Form should be completed**

→ **Satellite tag should be collected and send back to:** **TB Raab GmbH**
Quadenstraße 13
A-2232 Deutsch-Wagram, Austria

If carcass has been found contact local and regional/national police and report illegal circumstances. This step is very important in several cases (suspicion of illegal practices like poisoning and shooting). Police knows the legal situation among others i) to whom the found bird belongs, ii) who is allowed to decide about post-mortem examination/particular destination, iii) who is allowed to remove the carcass/evidences, iv) how to proceed officially and who has to initiate further steps. Both local and regional/national police should be informed to ensure that all information/evidences are secured (avoid/reduce corruption).
The exact procedure and handling can differ from country to country.

Please wait for police and do not remove carcass. Circumstances should be investigated regarding to illegal persecution and suspected bird crime (see below). A visual inspection of the terrain and the bird found, including a systematic ground search of the surrounding area should be carried out to look for evidences. Take photos of all abnormalities. At least a minimum of 100m radius around the location where the carcass was found should be searched. Specialists like veterinarians, conservation agencies and/or hunting agency can help to classify the circumstances correctly. Carcass and evidences should be collected appropriately, done best by police, and submit it to pathology lab for necropsy (depending on country, carried out by police or sender/finder). Complete **Case Form** and collect satellite tag.

Please follow the guidelines in case the carcass is collected by yourself.

- Always make sure that before the carcass is removed, enough pictures with a good quality and overview of the surrounding are made

- Always use gloves to protect yourself (poisoning, bacteria etc.)
- Put the bird a plastic bag (best with zipper), put, if possible cold packs around and wrap it in paper
- If you take it home, please put it in the cooler or freezer (depending on duration)
- You will get further instruction from the LIFE EUROKITE project

In cases legal situation does not allow to carry out a post-mortem examination (owner of bird does not allow post-mortem examination, police consider the death of the bird to be from natural causes / no suspicion of human persecution is considered, ect.), the cause of mortality cannot be defined. Complete Case Form and collect satellite tag.

If carcass has been found near a power line, electricity pole or a wind turbine please make sure that you have pictures of the infrastructure and if possible, a photo of the plaque with the identification number and company (if there is a plaque). If you know the concerned company (or can ask locals) note the name.

Evaluation of the found bird: Is the case suspicious?

Inspect the satellite tag/carcass as well as the surrounding very carefully without touching or removing something (if necessary, document all steps!). Suspected bird crime is often not easy to identify – keep that in mind and if possible, bring in specialist, they may help to classify the circumstances!

If you investigate the satellite tag focus especially on the straps. Do the straps have been cut off or has the knot loosened naturally? Very tiny details can be very important in suspected bird crimes.

In intoxicated birds in particular, little or no specific abnormalities can be detected. Is it the first bird found in this area? Is there more than one dead bird? Are there other dead animals? If you look in detail at the bird focus on the beak and nose region. Is there any blood or leftover feed? Pay attention to unnatural colour, sings of foreign substances, dead insects, unnatural position of wings or legs. Do you see injuries/blood on the carcass?

Do you find suspicious things in the surrounding area? E. g. traps, other dead birds or animals, (toxic) baits or eggs? Are there fresh footprints/trampled vegetation/bark injuries on trees/broken branches?

Keep in mind: Not only in cases of bird crime, also in cases birds die due to anthropogenic influences post-mortem examinations are recommended in every case of a found dead bird. The examination is really helpful i) to detect the actual cause of mortality, ii) to distinguish between bird crime, anthropogenic cause of death or natural cause of death, iii) to collect data about mortality reasons especially bird of prey populations have to deal with and iv) to earn information about the satellite tag itself, e.g. functionality, destructions and fixation).

LIFE EUROKITE Assessment Protocol: Mortality Protocol

Appropriate collection of carcasses:

Before touching anything, document coordinates and take photos!
Contact Police in suspicious cases and wait for them, that all necessary data and samples can be collected without missing details due to change the finding situation.
Complete Case Form!

Especially for court cases and if judicial evidences are found, police should collect samples.

Generally, carcasses and evidences are best collected in sealable bags. In case if suspected poisoned eggs are found, place them in containers which do not brake and can be tightly sealed to guarantee that nothing will leak. Take photos of all samples, especially in court cases, the photos are the only left information to collect for the database.

Labelling samples: Every bag and evidence have to bear a perfectly legible label with at least information containing:

- Date
- Species/evidence
- Number of samples
- Locality
- Name and contact information of sender/finder
- Case code (from the case form)

Transport of samples to pathology lab

Refrigerate carcass and organic samples and submit as soon as possible to pathology lab. Use specialized courier services (Austria/part of EU: medlog, TNT, ect., contact TB Raab/ the LIFE EUROKITE team if more information is needed). Be aware of special rules which have to be applied if certain diseases (e.g. bird flu) are present in the area.

→ **Please avoid freezing samples in case the bird can be transported to the pathology within 1-2 days!** Freezing and advances delay of carcasses and samples may lead to no results. Microbiological analyses, gross pathology and histology of organs and tissues are difficult to evaluate due to structural changes and destruction of tissue. **The carcasses should be refrigerated as soon as possible.**

→ **If it is not possible to submit carcasses immediately to a pathology lab for dissection (1-2 days maximum), however, freezing can prevent further autolysis and post-mortem examination can still be carried out at a later point in time with limited assessment.**

→ **Birds should be placed in sealable bags. Put 1-2 cool packs and paper in a paper box (ca. 50x30x20cm).**

Case Form

In every case the attached **Case Form** should be completed by the searcher (even if no carcass or tag could be found), the sender or finder of the satellite tag or bird as it documents all necessary information and what has been found. It focuses especially on information about the locality (including benchmarks and metric scales) and the findings, as well as which samples were collected, and which further steps were initiated. It is also possible to specify a presumed cause of death of the found bird.

LIFE EUROKITE Assessment Protocol: Mortality Protocol

Please fill in at least the yellow marked parts of the case form. This is very important for further investigation and to determine the reason of death.

Collect as much additional information as is deemed pertinent to the investigation, such as weather conditions, hunting management plan, predator-control authorisation, ect.

Take photos (ideally in colour) containing the following pictures: overview; macro of the dead bird/satellite tag/feathers/remains of body; surrounding (also power lines, wind turbines, electricity poles); evidences (on some occasions photography is the only way of conserving the evidence), seized objects and document all findings.

The Case form should be completed directly in field, at least at the same day and then send together with the taken photos to MEGEG/LIFE EUROKITE Manuel Wojta and Eva Indruchová via e-mail manuel.wojta@tbraab.at and eva.indruchova@tbraab.at as they document all cases.

<u>post-mortem examination at pathology lab</u>

Case Form should be handed out to the examining pathology lab to deliver all information about the case and especially the locality details.

The attached **Necropsy Form** should be filled out by the examining veterinarian and after completion send to MEGEG. It will guide the veterinarian throw all necessary steps of dissection and helps with the forensic workup.

It includes general details about the bird and a cascade of investigations (including radiography, gross pathology, histopathology, microbiology, toxicology).

It should be documented whether the submitted samples were sealed.

Samples collected for toxicological analysis should be freezed immediately to ensure they remain in proper conservation conditions. At least we recommend to preserve samples of brain, liver and crop/stomach for toxicological analysis in the future. They should be stored freezed in sealed bags.

After the documentation of all results of the examination and completed investigations, all information gathered from these two parts (**Case Form** and **Necropsy Form**) as well as the information gathered from the logger (if bird was tagged) will then lead to definite cause of death.

LIFE EUROKITE Project: Contacts

If you have any questions about the protocol or you need help please feel free to contact:

Manuel Wojta	TB Raab GmbH	+43 676 936 66 23	manuel.wojta@tbraab.at
Rainer Raab	TB Raab GmbH	+43 664 452 75 63	rainer.raab@tbraab.at
Hannah Böing	TB Raab GmbH	+43 660 230 73 99	hannah.boeing@tbraab.at

LIFE EUROKITE Project: Flow Chart (1)

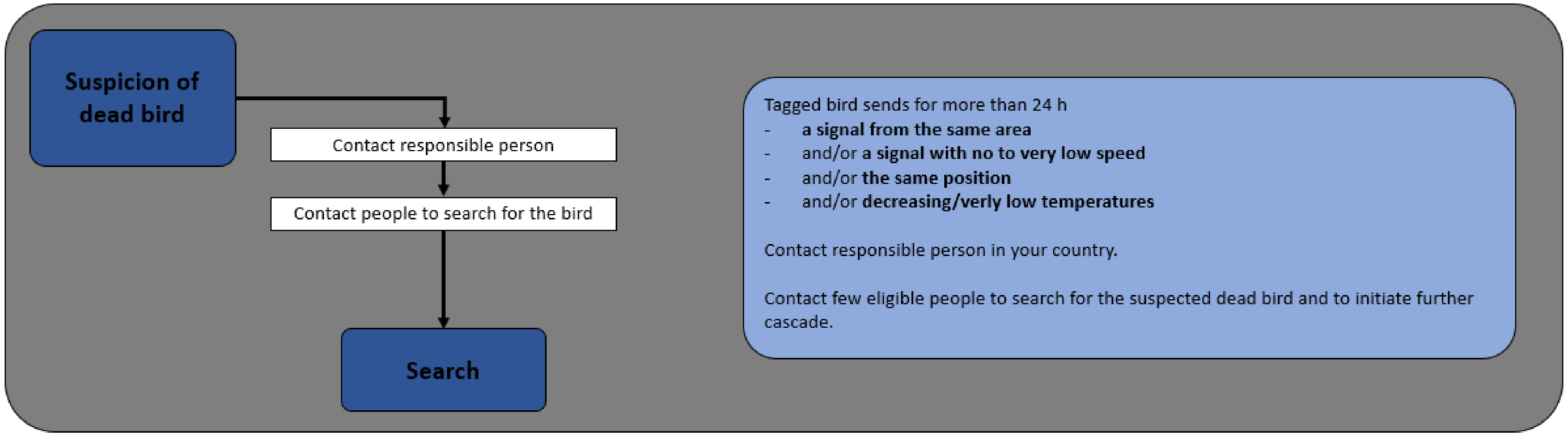

Tagged bird sends for more than 24 h

- **a signal from the same area**
- and/or **a signal with no to very low speed**
- and/or **the same position**
- and/or **decreasing/verly low temperatures**

Contact responsible person in your country.

Contact few eligible people to search for the suspected dead bird and to initiate further cascade.

LIFE EUROKITE Project: Flow Chart (2)

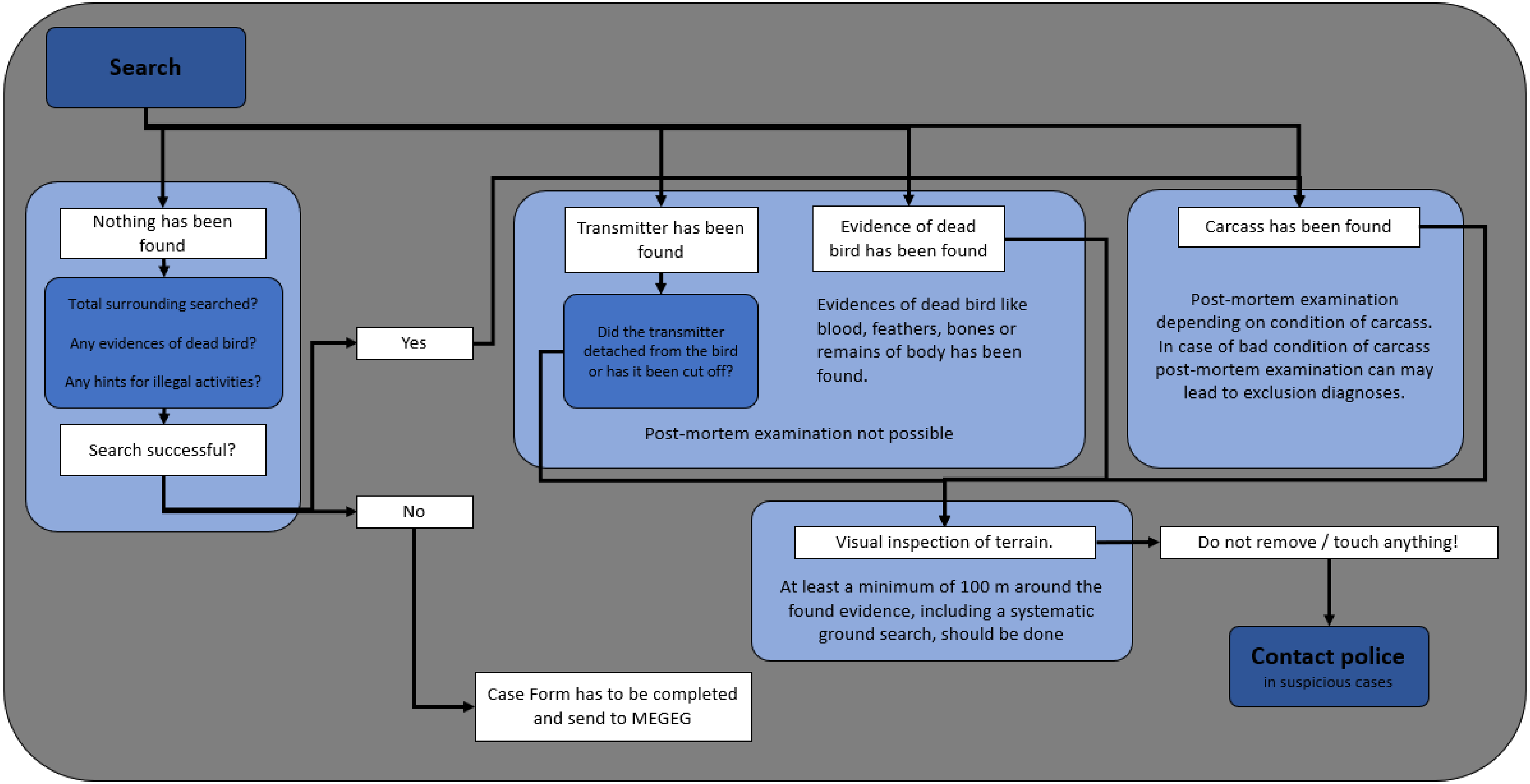

LIFE EUROKITE Project: Flow Chart (3)

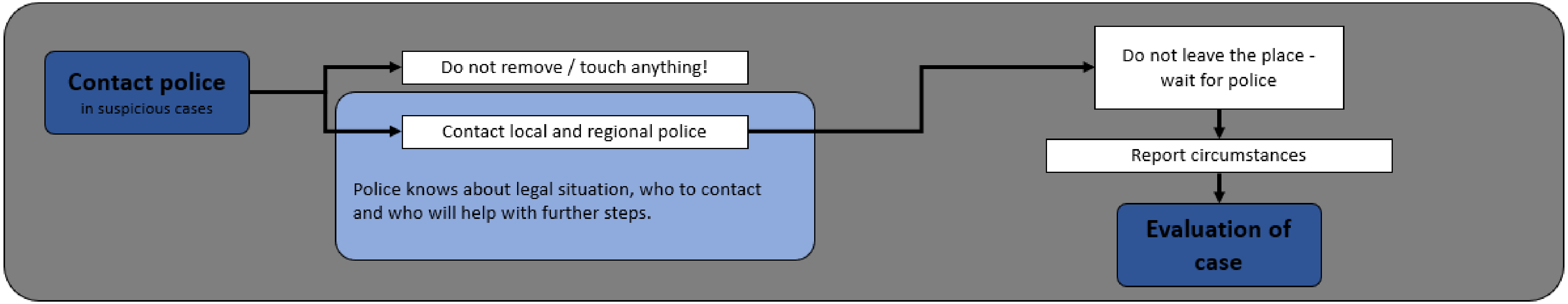

LIFE EUROKITE Project: Flow Chart (4)

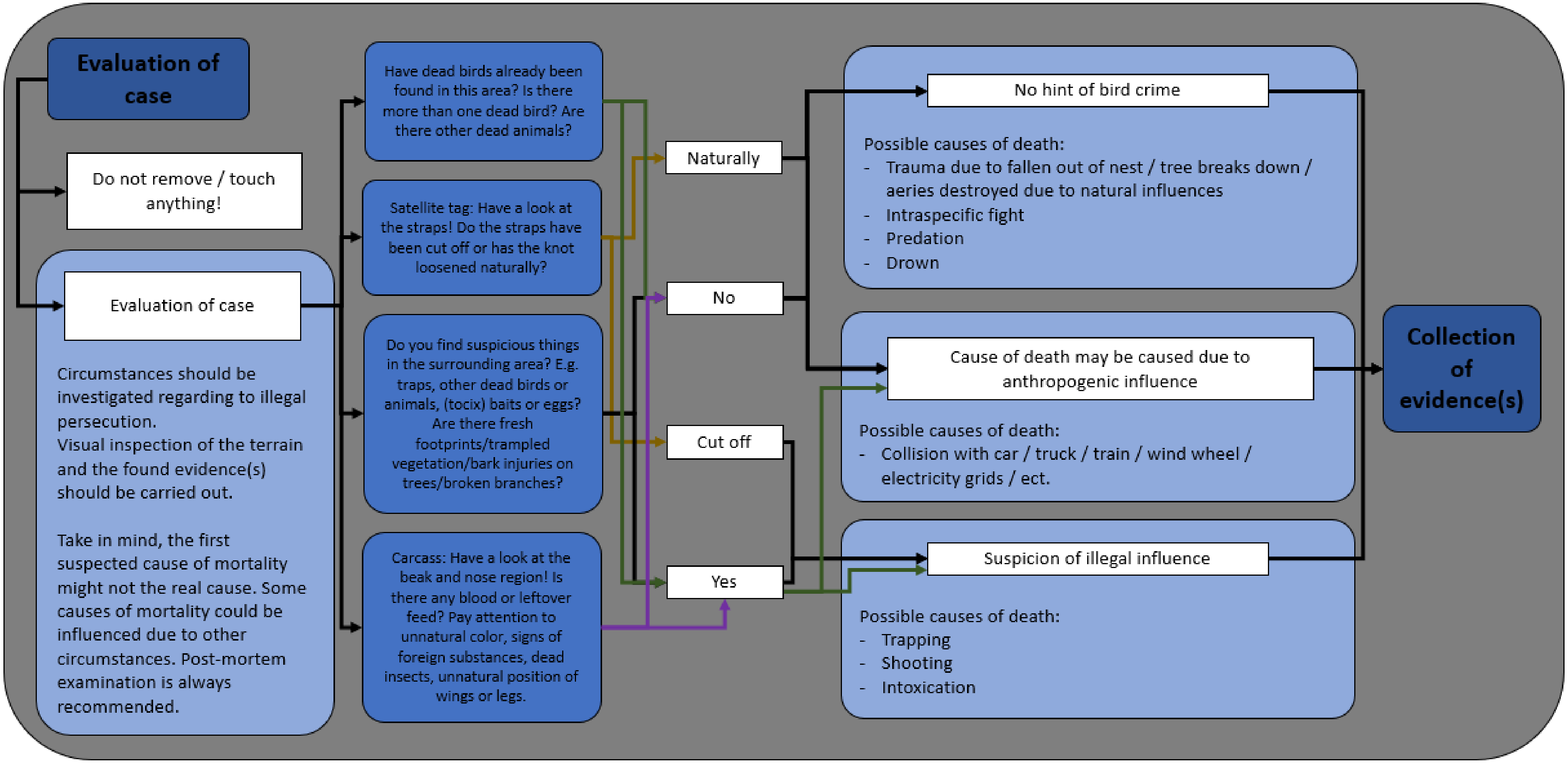

LIFE EUROKITE Project: Flow Chart (5)

Collection of evidence(s) → Collect satellite tag / Collect carcass / Collect evidence(s)

Collect satellite tag

Please leave satellite tag on the carcass as it can provide important and useful information during necropsy.

Collect carcass

Collect evidence(s)

→ **Case Form**

→ **Submit to pathology lab**

Appropriate collection of evidence(s) is best done by police, especially in court cases!

- Use sealable bags / containers which do not brake and can be tightly sealed
- All samples collected have to bear a perfectly legible label *(date, species / evidence, number of sample, locality, name and contact information of searcher, case code)*

Case Form → Assign case code, complete Case Form and send to MEGEG

Every case should be labelled consecutively:
DateOfSearch_CountryCode_ZipCode_SatelliteTagCode_NumberOfCaseForm

Take photos (overview, makro, surrounding) and send them to Manuel Wojta and Eva Indruchová via e-mail (manuel.wotja@tbraab.at, eva.indruchova@tbraab.at).
Document GPS coordinates.
All additional information can help to precise the cause of death of the found bird / evidence(s).

LIFE EUROKITE Project: Flow Chart (6)

Submit to pathology lab → Use appropriate transport → **Post-mortem examination**

Refrigerate carcass (do not freeze) and submit samples as soon as possible to pathology lab for post-mortem examination (within 1-2 days maximum). If the transport will take longer, please freeze the bird.

Use specialised courier services!

Post-mortem examination → Necropsy →

- Complete Necropsy Form and send to MEGEG / LIFE EUROKITE
- Return carcass to particular destination (disposal, taxidermist, etc.)

Case Form should be handed out to pathology lab, as it includes important information about case and locality details.

The information gathered from Case Form and Necropsy Form as well as the information from the GPS-logger (if bird was tagged) will then lead to a definitive cause of death.
