# Supplementary material for: A LEAP Forward in Wildlife Conservation: A Standardized Framework to Determine Mortality Causes in Large GPS‐Tagged Birds
Source: Ecol Evol. 2025 Mar 27;15(4):e70975. doi: 10.1002/ece3.70975 (PMC11949540; doi:10.1002/ece3.70975)
Supplement: Supplementary file 3 — Appendix S3. [file ECE3-15-e70975-s003.pdf]

LIFE EUROKITE Assessment Protocol: Necropsy Form

CASE CODE

____ _ ____ _ ____ _ ____ _ ____

DateOfSearch(YYYYMMDD)__CountryCode___CodeOfRegion_____SatelliteTagCode____ConsecutiveNumber

Date of completing: ____ / ____ / 20____ Examiner/Lab ID/Year: ____ / ____ /20____

Case Form received? ☐Yes ☐No Bird received on: ____ / ____ / 20____

Case code LIFE EUROKITE: ____

SAMPLES

Submitted samples sealed: ☐Yes ☐No

Samples/Species: ____

Identification markings/ring number: ____

Age: ☐Juvenile ☐Adult ☐Unknown ☐ ____

Weight: ____ g

Sex: ☐Male ☐Female ☐Unknown

Gonades: ☐Inactive ☐Active ☐Egg(s) in formation ☐Unknown

GENERAL INFORMATION

Condition of carcass: ☐Fresh ☐Good ☐Moderate ☐Bad ☐Frozen ☐Cannot be evaluated (cadaverous/mummified)

Body condition: ☐Fresh ☐Good ☐Moderate ☐Ill-nourished ☐Cachectic ☐Cannot be evaluated

PERFORMED DIAGNOSTICS

☐X-Rays ☐Gross pathology ☐Histology ☐Parasitology ☐Virology ☐Toxicology

☐Bacteriology/mycology ☐Other diagnostics: ____

Additional samples preserved ☐Yes ☐No

☐Heart ☐Lung ☐Liver ☐Spleen ☐Kidney ☐Skin ☐Brain

☐Stomach ☐Intestine ☐Other samples: ____

On behalf of: ____

X-RAYS

☐Done ☐Not done

Trauma – skeletal injuries

☐None ☐Head ☐Spine ☐Torso ☐Right wing ☐Left wing

☐Right leg ☐Left leg

Metal opacity / Evidence of gunshot

☐None ☐Head ☐Spine ☐Torso ☐Right wing ☐Left wing

☐Right leg ☐Left leg

Precise description:

____

Evaluation has to be combined with gross pathology!

GROSS PATHOLOGY

☐Done ☐Not done

Trauma: ☐Yes ☐No

Type of trauma: ☐Blunt ☐Pointed ☐Others:

Trauma extent: ☐Tissue ☐Bone

Location? ☐Head ☐Torso ☐Spine:
☐Right wing ☐Left wing ☐ Right leg ☐Left leg

Bleeding? ☐Trauma intravital ☐ Trauma postmortal

Precise description:

Evaluation: ☐Cause of death ☐Incidental finding ☐Evaluation not possible

Cause of trauma (Is location of carcass correlated to cause of death?):

Carcass

Abnormalities: ☐Trauma listed above ☐No abnormality detected

☐Coat:

☐Skin

☐Orifices of the body

☐Eyes

☐Nose and beak

☐Ears

☐Additional information:

Musculoskeletal and central nervous system

Abnormalities: ☐Trauma listed above ☐No abnormality detected

☐Skeleton

☐Muscles

☐Brain

☐Nervs

☐Additional information:

GROSS PATHOLOGY – visceral cavity and organs ☐Done ☐Not done

Respiratory system

Abnormalities: ☐Trauma listed above ☐No abnormality detected

☐Trachea/ Lung/ Air sacs

☐Additional information:

Circulation

Abnormalities: ☐Trauma listed above ☐No abnormality detected

☐Heart/Vessels

☐Additional information:

Parenchyma

Abnormalities: ☐Trauma listed above ☐No abnormality detected

☐Pancreas

☐Liver

☐Spleen

☐Additional information:

Urinary tract

Abnormalities: ☐Trauma listed above ☐No abnormality detected

☐Kidney

☐Additional information:

Digestive tract

Abnormalities: ☐Trauma listed above ☐No abnormality detected

☐Oesophagus/Crop

Filling: ☐Well ☐Moderate ☐Mild ☐Poorly ☐Empty

Content: ☐Pellet ☐Bones ☐Muscels ☐Others:
☐Cannot be evaluated

☐Stomach

Filling: ☐Well ☐Moderate ☐Mild ☐Poorly ☐Empty

Content: ☐Pellet ☐Bones ☐Muscels ☐Others:
☐Cannot be evaluated

☐Intestinal tract

Filling: ☐Well ☐Moderate ☐Mild ☐Poorly ☐Empty

Content: ☐Cannot be evaluated

☐Additional information

BACTERIOLOGY/MYCOLOGY

☐Done ☐Not done

Examinded organ(s): ☐Lung ☐Liver ☐Kidney ☐Skin ☐Intestine
☐Other sample(s):

Spread infection: ☐Local ☐Generalized

Diagnostic findings:

Portal of entry: ☐Organ(s): ☐Injury:
☐Unknown

Evaluation: ☐Cause of death ☐Incidental finding ☐Evaluation not possible

☐Additional information:

VIROLOGY

☐Done ☐Not done

Examinded organ(s): ☐Lung ☐Liver ☐Spleen ☐Skin ☐Intestine
☐Other sample(s):

Spread infection: ☐Local ☐Generalized

Diagnostic findings:

Evaluation: ☐Cause of death ☐Incidental finding ☐Evaluation not possible

☐Additional information:

PARASITOLOGY

☐Done ☐Not done

Ectoparasites: ☐Negative ☐Mild ☐Moderate ☐Severe

☐Mites ☐Nits ☐Louse flies ☐Ticks ☐Fleas
☐Others:

Endoparasites: ☐Negative ☐Mild ☐Moderate ☐Severe

☐Capillaria ☐Ascaridia ☐Cestodes ☐Coccidia ☐Trichomonads
☐Others:

Proven parasites in histology: ☐Negative ☐Mild ☐Moderate ☐Severe

In: ☐Heart ☐Lung ☐Liver ☐Spleen ☐Kidneys
☐Muscle ☐Skin ☐Brain
☐Intestinal tract: ☐Others:

Evaluation: ☐Cause of death ☐Incidental finding ☐Evaluation not possible

☐Additional information:

LIFE EUROKITE Assessment Protocol: Necropsy Form

TOXICOLOGY

☐Done ☐Not done

Sample: ☐Content of crop/oesophagus ☐Content of stomach ☐Liver
☐Others:

Evidence: ☐No substances detected
☐Cholinesterase inhibitors (carbofuran) ☐Anticoagulants/Coumarin derivate ☐Lead
☐Expanded toxicological screening:

Evaluation: ☐Cause of death ☐No definite interpretation possible
☐Subclinical value/incidental finding

☐Additional information:

HISTOLOGY

☐Done ☐Not done

☐Heart

☐Lung

☐Liver

☐Kidney

☐Spleen

☐Stomach/Intestine

☐Brain

☐Other samples

Proven inflammation in histology: ☐Yes ☐No

Affected organ(s): ☐Heart ☐Lung ☐Liver ☐Spleen ☐Kidney
☐Skin ☐Brain ☐Stomach ☐Intestine
☐Other sample:

Cause of inflammation: ☐Bacteria ☐Fungi ☐Viruses ☐Parasites
☐Others:

Evaluation: ☐Cause of death ☐Incidental finding ☐Evaluation not possible

☐Additional information:

DIAGNOSIS
